# Supplementary material for: Electrically reconfigurable extended lasing state in an organic liquid-crystal microcavity
Source: Nat Commun. 2026 Apr 16;17:5335. doi: 10.1038/s41467-026-71733-0 (PMC13272646; doi:10.1038/s41467-026-71733-0)
Supplement: Supplementary file 1 — Supplementary Information [file 41467_2026_71733_MOESM1_ESM.pdf]

Supplemental information: Electrically Reconfigurable Extended Lasing State in an Organic Liquid-Crystal Microcavity

Dmitriy Dovzhenko[1*], Luciano Siliano Ricco[2,3], Krzysztof Sawicki[1,4], Marcin Muszyński[3], Pavel Kokhanchik[5], Piotr Kapuściński[3], Przemysław Morawiak[6], Wiktor Piecek[6], Piotr Nyga[7], Przemysław Kula[8], Dmitry Solnyshkov[5,9], Guillaume Malpuech[5], Helgi Sigurðsson[3], Jacek Szczytko[3], Simone De Liberato[1,10]

[1]School of Physics and Astronomy, University of Southampton, University Road, Southampton, SO17 1BJ, United Kingdom.
[2]Science Institute, University of Iceland, Dunhagi-3, Reykjavik, IS-107, Iceland.
[3]Institute of Experimental Physics, Faculty of Physics, University of Warsaw, ulica Pasteura 5, Warsaw, PL-02-093, Poland.
[4]Department of Physics, Durham University, South Road, Durham, DH1 3LE, United Kingdom.
[5]Institut Pascal, Université Clermont Auvergne, CNRS, ClermontINP, Clermont-Ferrand, F-63000, France.
[6]Institute of Applied Physics, Military University of Technology, S. Kaliskiego 2, Warsaw, 00-908, Poland.
[7]Institute of Optoelectronics, Military University of Technology, S. Kaliskiego 2, Warsaw, 00-908, Poland.
[8]Institute of Chemistry, Military University of Technology, S. Kaliskiego 2, Warsaw, 00-908, Poland.
[9]Institut Universitaire de France, Paris, F-75231, France.
[10]Istituto di Fotonica e Nanotecnologie, Consiglio Nazionale delle Ricerche (CNR), Piazza Leonardo da Vinci 32, Milano, 20133, Italy.

*Corresponding author(s). E-mail(s): dovzhenkods@gmail.com;

Supplementary Note 1. Individual lasing state emission properties

We note that below the threshold, despite the vertical polarisation of the excitation, we observed emission from both horizontal and vertical cavity modes due to the ultrafast intermolecular energy transfer mechanism responsible for the depolarisation of the photoluminescence (PL) in films of organic laser dyes with similar structure and properties [1, 2]. In contrast, when pump power exceeds the threshold, we observed a sharp increase of the degree of linear polarisation with the polarisation of the lasing state inherited from the polarisation of the pump, see Supplementary Fig. 1. Here, we excited an individual lasing state using a linearly polarized pump in the regime of small TE-TM splitting near $k_{\|} = 0$, where the difference in energy of the emission above the lasing threshold for horizontal and vertical polarizations remains negligible and gain-induced anisotropy defines the polarization inheritance. Notably, apart from the gain-induced anisotropy allowing for the control of the emission polarization above the threshold, cavity anisotropy can be introduced as a competing mechanism under specific detuning conditions. In the case of a large relative spectral detuning between the horizontal and vertical cavity modes, the gain difference can become strong enough to favor a particular polarization of the emission such that the linear polarization of the emission above the threshold can be perpendicular to the polarization of the pump. In the experiments presented in the current study, we avoided such a regime of operation. Photostability of the emission was tested in the multi-shot regime of the excitation, with a 20 Hz repetition rate and a 5 ns pulse length. No apparent degradation of the lasing was observed for more than 10 minutes of excitation, yielding more than 12,000 laser pulses generated.

Supplementary Fig. 2 shows characteristics of the PL for a single isolated lasing state around Rashba-Dresselhaus (RD) regime of spin-orbit coupling (SOC). In Supplementary Fig. 2(a,b) we show energy-resolved momentum space PL below the lasing threshold measured (a) exactly in RD SOC regime at 1840 mV and (b) in slightly detuned regime at 1880 mV external voltage. We further demonstrate the polarisation properties the emission in each regime providing momentum-space distribution of the non-normalised S_3 (Supplementary Fig. 2(c)) and S_1 (Supplementary Fig. 2(d)) Stokes components of the lasing state.

Supplementary Note 2. Spatial coherence in a dyad supermode

To further support the claim of phase-locking between the spatially separated lasing states and electrical tunability of such effect we performed spatially resolved interferometry measurements for the case of dyad supermode with the spot separation distance of 13 μm, Supplementary Fig. 3. Single shot interferometry measurements were made using Mach-Zehnder interferometer at 0 time delay to spatially overlap the real space emission from the opposite lasing states of a dyad (see schematic in Supplementary Fig. 3). We observed clear interference fringes for 0 V (Supplementary Fig. 3(a)), highlighting the synchronization between the states. Increase of the voltage

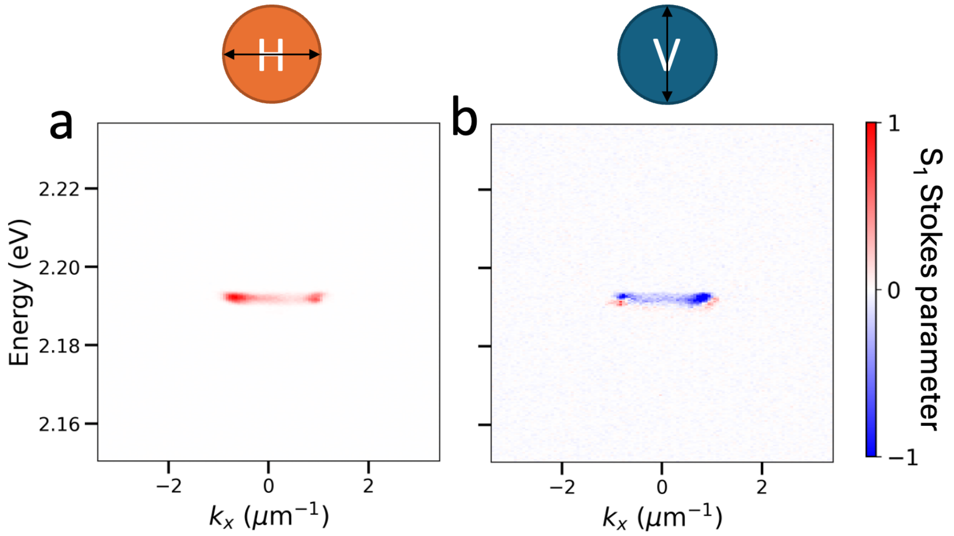

Supplementary Fig. 1 Inheritance of the lasing state polarisation. **(a,b)** Energy-resolved along $k_y = 0$ momentum-space distribution of the non-normalised S_1 Stokes component of the emission from a single spot pumped with **(a)** horizontally (H) and **(b)** vertically (V) polarised light. Schematic above shows the respective polarisation of the pump

to 1.6 V was accompanied by the loss of fringes visibility corresponding to desynchronisation, while further increase to 1.84 V showed revival of the fringes visibility and re-establishing of the phase-locking in a dyad. We therefore prove that the appearance of the fringe pattern in the real- and momentum-space of the emission from the array of spatially extended lasing states is accompanied by a long-range phase-coherence.

Supplementary Note 3. Additional data on coupling in the presence of spin-orbit interaction

We state in Sec. 2.3 of the main manuscript that we observed the revival of the coupling between separated lasing states in a dyad configuration when the system enters the RD SOC regime. However, while the signature fringe pattern is clearly observed in Fourier-space and further evidenced by the coherence revival in interferometry data (see Supplementary Note 2), its visibility is lowered in the real-space image of total emission. We attribute this effect to two contributing factors stemming from the change of the Fourier space emission pattern in the presence of spin-orbit coupling. Firstly, due to the spin-dependent redistribution of the emission in Fourier space, instead of two counter-propagating waves of the same polarization, interfering constructively or destructively to form a pronounced pattern, two phase-shifted waves of opposite polarization are present, which do not interfere directly. The consecutive spatial shift between the emission patterns of opposite circular polarisation makes fringe pattern

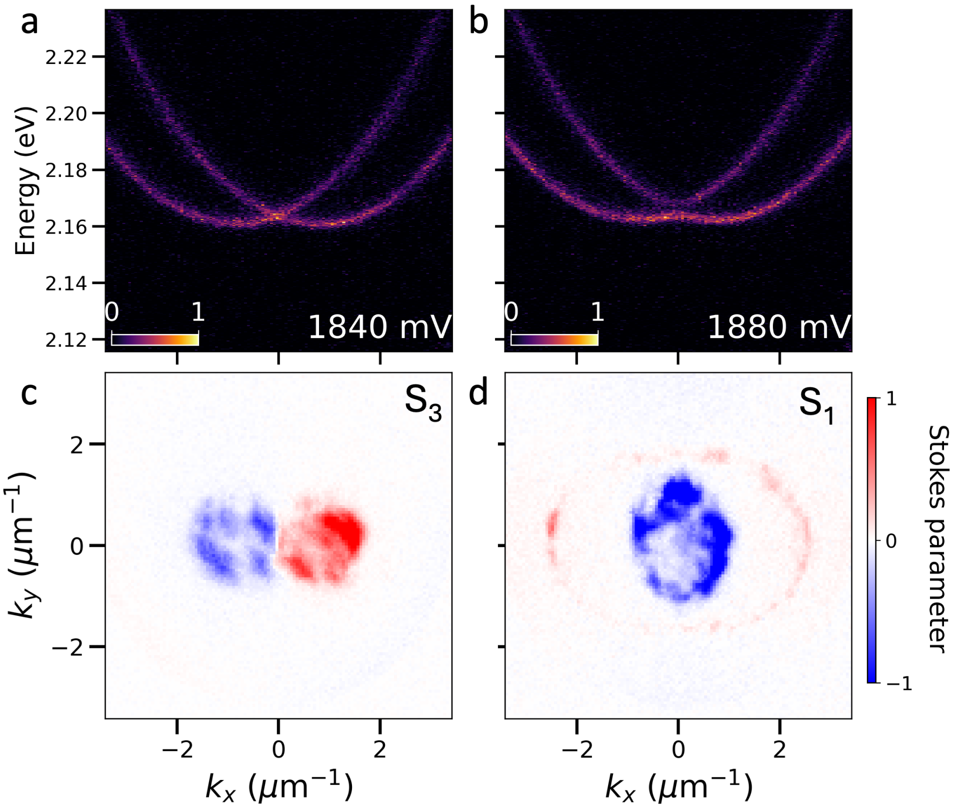

Supplementary Fig. 2 Emission characteristics of a single lasing state. **(a,b)** Energy-resolved momentum-space along $k_y = 0$ for two values of external voltage, corresponding to **(a)** 1840 mV and **(b)** 1880 mV pumped below $(0.5P_{th})$ the lasing threshold and **(c,d)** corresponding momentum-space distribution of the non-normalised S_3 **(c)** and S_1 **(1)** Stokes components of the lasing state pumped above $(1.5P_{th})$ the lasing threshold. Colour scales represent normalized **(a,b)** photoluminescence intensity and **(c,d)** Stokes parameter in arbitrary units.

in the real-space total intensity distribution (Fig.5(c) in the main text) drastically reduced. In Supplementary Fig. 4 we show the corresponding experimentally measured polarisation-resolved real-space distribution of the emission from a coupled dyad state in the RD SOC regime at an 1840 mV external voltage. Characteristic fringe pattern is observed for both polarisation components. We note that the visibility of the fringes in polarisation-resolved images remains lower than that typically observed for a linearly polarized dyad, which can be attributed to the second consequence of the RD SOC regime. Both circular polarization components have a dominant direction of propagation with σ^+ polarised emission propagating from the left lasing spot to the right and σ^- polarised emission vice versa with only a small fraction of the intensity propagating in the opposite direction [as follows from their respective distributions in

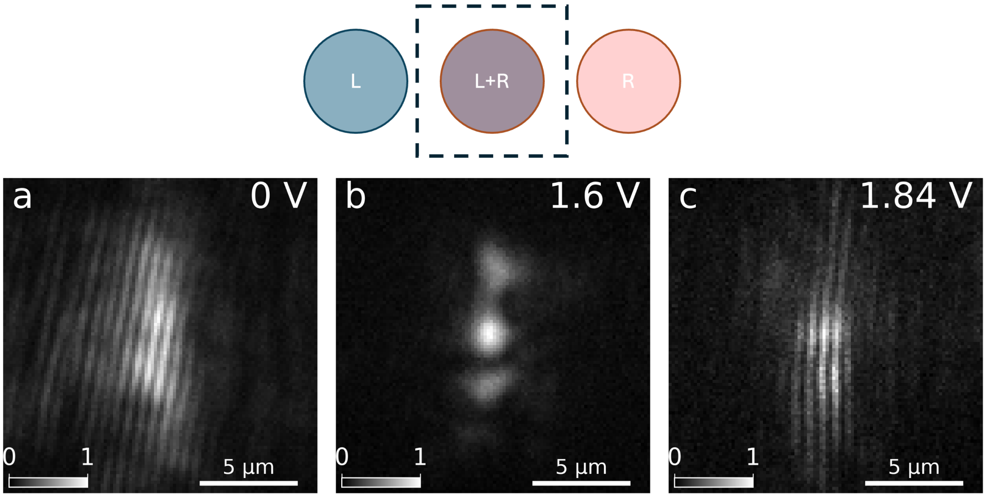

Supplementary Fig. 3 Spatially extended coherence. Experimental interferogram of the dyad supermode obtained by overlapping the real-space emission of the opposite lasing states of a dyad going through the separate arms of Mach-Zehnder interferometer at **(a)** 0 V, **(b)** 1.6 V, **(c)** 1.84 V. Schematic above represents the overlapping real-space emission of a dyad (left and right circles) from the different (red and blue) arms of the interferometer with the region depicted in images below shown by dashed square. Colour scales represent normalized photoluminescence intensity (arbitrary units).

Fourier space]. As a result, while phase-locking is still achieved by directional propagation of the emission components between the lasing spots, each of the polarisation components between the lasing spots does not have a counter-propagating wave of similar intensity and polarisation to form a strong interference pattern with. The same effect of limited fringes visibility and low total intensity between the lasing spots at 1840 mV is clearly present in numerical simulations (Fig. 6 of the main manuscript). Once the polarization-resolved Fourier-space distribution of the emission from each lasing state becomes symmetric again relative to the k_y axis (1880 mV voltage case in Fig.6(c) in the main text) the interference fringes between the lasing spots in the real space distribution of the total emission are gradually restored.

Supplementary Note 4. Additional data on unconventional coupling

In Sec. 2.4 of the manuscript we demonstrate realisation of next-nearest-neighbour (NNN) coupling regime by analysing the periodicity of interference fringes visible in momentum space. In Supplementary Fig. 5 we show polarisation resolved PL in momentum space for NNN regime of interaction corresponding to the Fig.7(f) of the main manuscript. Phase-locking is manifested between horizontally polarised states, while no interference fringes are observed in vertically polarised emission.

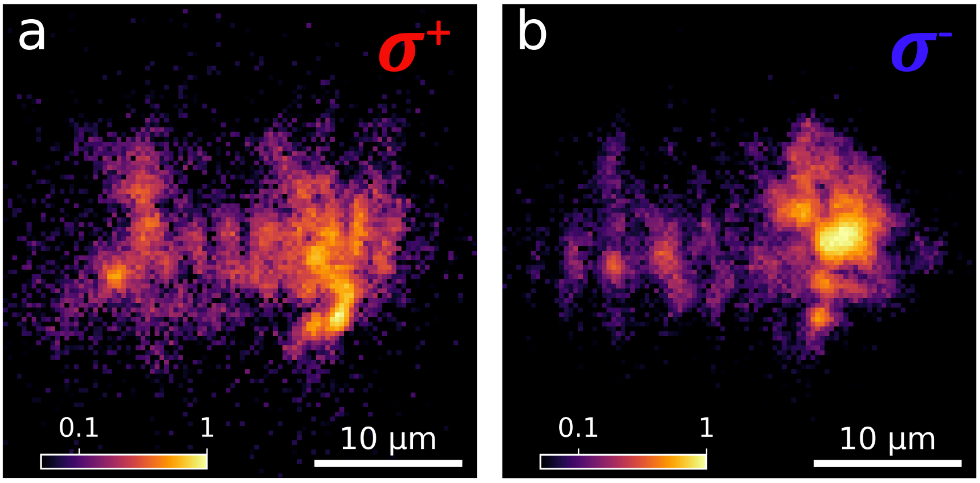

Supplementary Fig. 4 Dyad lasing state in the presence of spin-orbit coupling. **(a)** Right-circularly polarised (σ^+) and **(b)** Left-circularly polarised (σ^-) polarised real-space distribution of the emission for two pump spots at 1840 mV showing coupled supermode lasing state. Colour scales represent normalized photoluminescence intensity (arbitrary units). For better visibility images are illustrated in logarithmic scale.

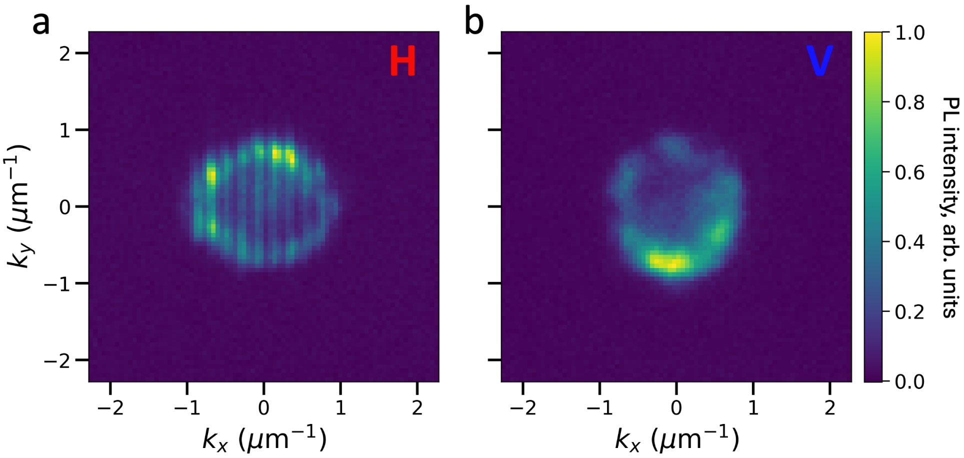

Supplementary Fig. 5 Next-nearest-neighbour coupling regime. Horizontally **(a)** and vertically **(b)** polarised momentum-space distribution of the emission from 3 lasing states in the NNN coupling regime.

Supplementary Note 5. Determination of the coupling by extracting photonic component

The standard way of strong coupling determination is the observation of cavity mode anticrossing while the photonic mode approaches the exciton. In our sample, any

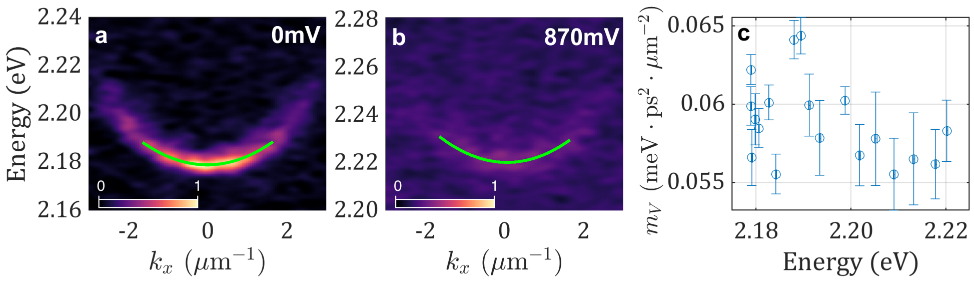

Supplementary Fig. 6 Experimental dispersion of the tunable vertically-polarized cavity mode below the lasing threshold for the voltage of (a) 0 mV and (b) 870 mV; the green line demonstrates the fitting by a parabola; (c) extracted mode mass for different voltages by fitting with a parabolic function; error bars represent standard error of the fit and do not include experimental uncertainties; the mode mass does not increase while approaching the excitonic resonance, therefore, the mode is in the weak coupling regime. Colour scales represent normalized photoluminescence intensity (arbitrary units).

photonic mode has a parabolic shape and does not demonstrate anticrossing. More precisely, any mode vanishes before we start seeing anticrossing both at the high wavevector or at the low wavevector when it is shifted towards the exciton, which is applicable for tunable vertically polarized modes. However, as it has been shown in a recent study [3], when the exciton resonance is widely inhomogeneously broadened, the absence of anticrossing does not signify the absence of strong coupling for high exciton-photon detunings. A more detailed analysis of modes is needed, which we perform in the following.

We follow a tunable vertically polarized photonic mode for different values of applied voltage until it vanishes due to the proximity to the exciton located at 2.4 eV for the dye P580. For each value of voltage, we fit the mode with a parabola, as shown in Supplementary Fig. 6(a,b) for two values of voltage (all dispersions for voltage values in-between demonstrate a good fit by a parabola, as well with a coefficient of determination $R^2 > 0.96$). We extract the effective mass and the bottom (mode) energy of each parabola and plot them together in Supplementary Fig. 6(c). In the case of the strong coupling, this dependence has to show a gradual increase of the mode mass when approaching the exciton [3]. In our case, we see that the mass remains approximately constant. Therefore, we conclude that the sample is in the weak coupling regime.

Supplementary Note 6. Additional Simulations using the Non-Hermitian 2D Schrödinger Model

This section presents additional data for simulated real and Fourier-space distributions using the Non-Hermitian 2D Schrödinger equations (NHSE) as described in section 4.3 of the main text. Particularly, in Supplementary Fig. 7 we show the simulations for the dyad supermode lasing states as a function of the distance between pump spots, corresponding to cases depicted in Fig. 3 of the main text. We also depict numerical

simulations for the case of unconventional coupling in the 1D chain of pump spots as seen in Supplementary Fig. 8, corresponding to experimental profiles from Fig. 7 in the main text.

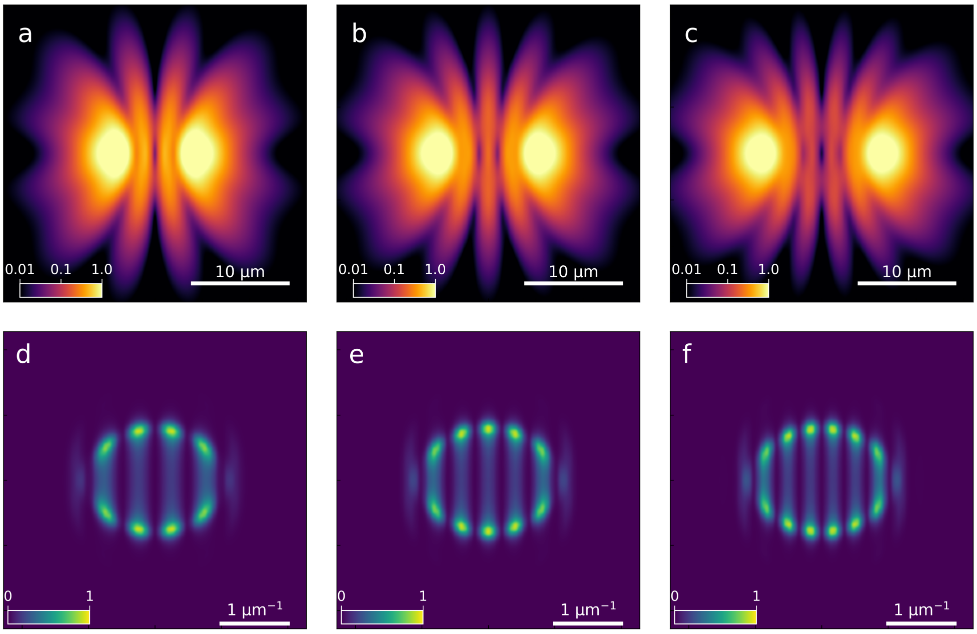

Supplementary Fig. 7 Simulation for the dyad supermode lasing state for distinct distances d between pump spots, corresponding to profiles shown in Fig. 3 of the main text. Panels **(a,d)** exhibit real and Fourier-space profiles for $d = 9\,\mu\text{m}$, while **(b,e)** and **(d,f)** correspond to $d = 11\,\mu\text{m}$ and $d = 13\,\mu\text{m}$, respectively. Colour scales represent normalized photoluminescence intensity (arbitrary units). For better visibility real-space images (a-c)are illustrated in logarithmic scale saturated below 0.01.

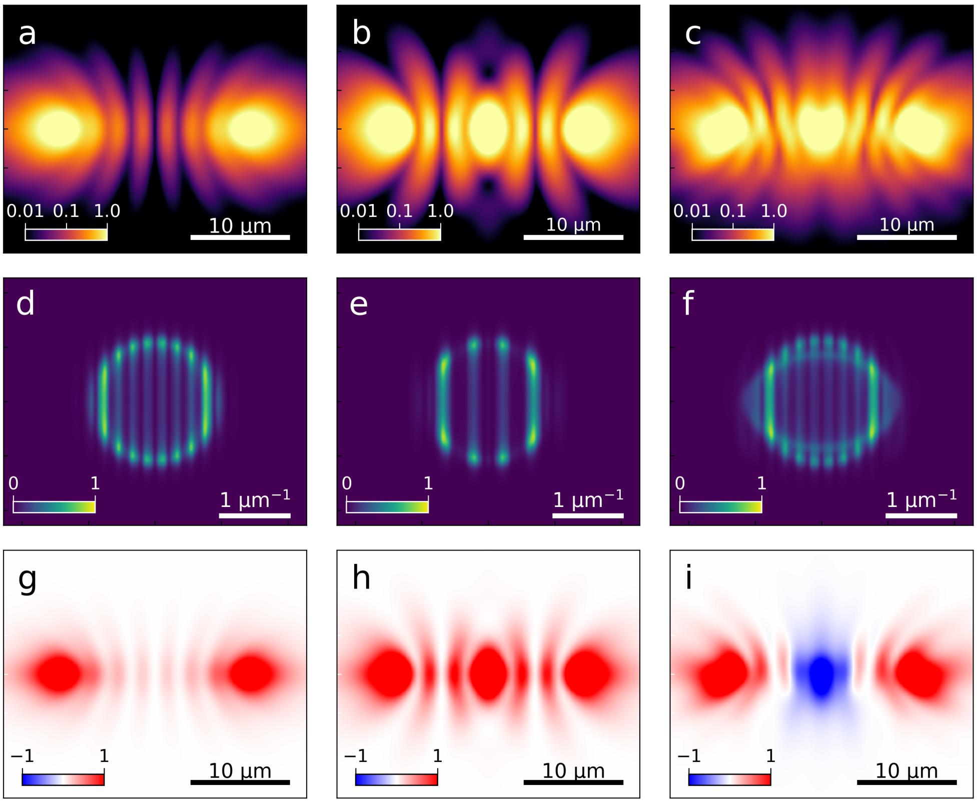

Supplementary Fig. 8 Simulation for an unconventional coupling in a 1D chain of pump spots corresponding to emission profiles shown in Fig. 7 of the main text. The top and middle panels show the numerical simulations for both real and Fourier-space profiles, with the bottom panels exhibiting the real-space $S_1(\boldsymbol{r})$ component of the Stokes vector describing the degree of linear polarisation of the emission profile as defined in Eq. (3) of the main text. **(a,d,g)** describes two vertically polarised pump spots at 20 µm separation distance. **(b,e,h)** show the simulations for three vertically polarised pump spots separated by 10 µm from each other. **(c,f,i)** also numerically describes three pump spots separated by 10 µm, but with the central spot pumped with horizontally polarised light. Colour scales represent normalized **(a-f)** photoluminescence intensity and **(g-i)** S_1 Stokes parameter (arbitrary units). For better visibility real-space images (a-c)are illustrated in logarithmic scale saturated below 0.01.

Supplementary References

[1] Chang, M.H., Frampton, M.J., Anderson, H.L., Herz, L.M.: Intermolecular Interaction Effects on the Ultrafast Depolarization of the Optical Emission from Conjugated Polymers. Physical Review Letters **98**(2), 027402 (2007) https://doi.org/10.1103/PhysRevLett.98.027402

[2] Musser, A.J., Rajendran, S.K., Georgiou, K., Gai, L., Grant, R.T., Shen, Z., Cavazzini, M., Ruseckas, A., Turnbull, G.A., Samuel, I.D.W., Clark, J., Lidzey, D.G.: Intermolecular states in organic dye dispersions: excimers vs. aggregates. Journal of Materials Chemistry C **5**(33), 8380–8389 (2017) https://doi.org/10.1039/C7TC02655B

[3] Muszynski, M., Kokhanchik, P., Urbonas, D., Kapuscinski, P., Oliwa, P., Mirek, R., Georgakilas, I., Stoferle, T., Mahrt, R.F., Forster, M., et al.: Observation of a stripe phase in a spin-orbit coupled exciton-polariton bose-einstein condensate. arXiv preprint arXiv:2407.02406 (2024)
