# Supplementary material for: Electrically reconfigurable extended lasing state in an organic liquid-crystal microcavity
Source: Nat Commun. 2026 Apr 16;17:5335. doi: 10.1038/s41467-026-71733-0 (PMC13272646; doi:10.1038/s41467-026-71733-0)
Supplement: Supplementary file 2 — Reporting Summary [file 41467_2026_71733_MOESM2_ESM.pdf]

nature research

Corresponding author(s): Dovzhenko Dmitriy

Lasing Reporting Summary

Nature Research wishes to improve the reproducibility of the work that we publish. This form is intended for publication with all accepted papers reporting claims of lasing and provides structure for consistency and transparency in reporting. Some list items might not apply to an individual manuscript, but all fields must be completed for clarity.

For further information on Nature Research policies, including our data availability policy, see Authors & Referees.

ü Experimental design

Please check: are the following details reported in the manuscript?

1. Threshold

Item	Yes/No	Details
Plots of device output power versus pump power over a wide range of values indicating a clear threshold	[x] Yes [ ] No	Sec. 2.1 of the main text, Fig. 2(f)

2. Linewidth narrowing

Item	Yes/No	Details
Plots of spectral power density for the emission at pump powers below, around, and above the lasing threshold, indicating a clear linewidth narrowing at threshold	[x] Yes [ ] No	Sec. 2.1 of the main text, Fig.2(a-d, f)
Resolution of the spectrometer used to make spectral measurements	[x] Yes [ ] No	Sec. 4.2 of the main text

3. Coherent emission

Item	Yes/No	Details
Measurements of the coherence and/or polarization of the emission	[x] Yes [ ] No	Coherence: Supplementary Note 2, Supplementary Fig. 3. Polarization: Supplementary Note 1, Supplementary Fig.1.

4. Beam spatial profile

Item	Yes/No	Details
Image and/or measurement of the spatial shape and profile of the emission, showing a well-defined beam above threshold	[x] Yes [ ] No	Sec. 2.2 and 2.3 of the main text, Fig. 3 and Fig. 4

5. Operating conditions

Item	Yes/No	Details
Description of the laser and pumping conditions *Continuous-wave, pulsed, temperature of operation*	[x] Yes [ ] No	Sec. 4.2 of the main text, 1st paragraph
Threshold values provided as density values (e.g. W cm^{-2} or J cm^{-2}) taking into account the area of the device	[x] Yes [ ] No	Threshold energy of the excitation pulse can be found in Sec. 2.1 of the main text, 3d paragraph. Area of the device depends on the geometry of the pump. Typical single pumping spot size is provided in Sec. 4.2 of the main text, 1st paragraph.

6. Alternative explanations

Item	Yes/No	Details
Reasoning as to why alternative explanations have been ruled out as responsible for the emission characteristics *e.g. amplified spontaneous, directional scattering; modification of fluorescence spectrum by the cavity*	[x] Yes [ ] No	Alternative explanation by polaritonic condensation is ruled out in the Supplementary Note 5

7. Theoretical analysis

Item	Yes/No	Details
Theoretical analysis that ensures that the experimental values measured are realistic and reasonable *e.g. laser threshold, linewidth, cavity gain-loss, efficiency*	[x] Yes [ ] No	Theoretical model is presented in Sec. 4.3 of the main text and Supplementary Note 6, numerical simulation results are shown in Fig. 6 of the main text, Supplementary Fig. 7 and Supplementary Fig. 8.

8. Statistics

Item	Yes/No	Details
Number of devices fabricated and tested	[ ] Yes [x] No	Each lasing spot effectively constitutes a separate lasing device, the reported supermode lasing was clearly observed across the surface area of the few samples but rigorous statistical analysis is beyond the scope of the first proof-of-concept demonstration.
Statistical analysis of the device performance and lifetime (time to failure)	[x] Yes [ ] No	Statistics of the device performance provided in the Supplementary Note 1
