# Supplementary material for: Electrically reconfigurable extended lasing state in an organic liquid-crystal microcavity
Source: Nat Commun. 2026 Apr 16;17:5335. doi: 10.1038/s41467-026-71733-0 (PMC13272646; doi:10.1038/s41467-026-71733-0)
Supplement: Supplementary file 3 — Transparent Peer Review file [file 41467_2026_71733_MOESM3_ESM.pdf]

nature portfolio

Peer Review File

Electrically Reconfigurable Extended Lasing State in an Organic Liquid-Crystal Microcavity

Corresponding Author: Dr Dmitriy Dovzhenko

This file contains all reviewer reports in order by version, followed by all author rebuttals in order by version.

Version 0:

Reviewer comments:

Reviewer #1

(Remarks to the Author)
The manuscript by Dovzhenko et al. reports on the realization of spatially extended lasing "supermodes" in an organic liquid-crystal microcavity, operating in the weak coupling regime at room temperature. The authors demonstrate electrical control over the coupling between optically pumped lasing spots and the emergence of phase-locked extended emission patterns. Despite the blueshift of the emission stops once lasing locks to the cavity mode, which is the signature of lasing in the weak coupling regime, this is sufficient to enable a coherent outflow of photons that mediates coupling between different spatially separated lasing spots.

Overall, the experimental work is well done, and the topic is of potential interest to the community interested on reconfigurable photonic devices for which working at room temperature is essential. However, while the results are sound, the novelty compared to existing literature is not entirely clear and should be discussed more explicitly. In particular, similar concepts—both in inorganic (III–V, II–VI) systems and in recent room-temperature polaritonic platforms—have been reported. The authors should better position their work with respect to prior results. The demonstration of coupled lasing states and "supermodes" recalls several works in III–V polariton systems, and more recently also in room-temperature materials. For instance, Tao et al., Nat. Mater. 21, 761 (2022) [Ref. 24] already showed room-temperature polaritonic XY spin Hamiltonians with very nice data.

Likewise, the concept of electrically controlled polarization switching in hybrid or liquid-crystal cavities has been addressed by the same authors using the same system (see Ref. 41).

The manuscript currently lists these references under general discussions putting them here and there (e.g., "spin simulation"), but it should explicitly acknowledge what is new here beyond those achievements.

More specific comments:

1. The term supermode is used even when a single spot is lasing. The authors should clarify what they mean by "supermode". Conventionally, a supermode implies coherent coupling between at least two sub-modes; in which sense this is valid here?

2. Figures 3 and 4 appear to be referred to in reverse order in the text. Please check the sequence and ensure consistent references throughout the manuscript.

3. The role of the pump polarization is not fully clear. The authors state that "the polarization of the lasing state is inherited from the pump polarization," but the presented data seem instead to indicate that the emission polarization follows the cavity eigenmode that lases at threshold. For instance, in Fig. 1(f), the emission appears vertically polarized (parallel to the lower-energy cavity mode). Later, for three-spot configurations, the emission polarization can be tuned by changing the pump polarization. Does this tuning also shift the emission energy? The authors should clarify whether the lasing state polarization is dictated by the cavity anisotropy or by pump-induced gain anisotropy, and discuss whether both effects compete.

4. It would be helpful to explicitly show the small blueshift residual for the 1600 mV case, where no ballistic expansion is observed (e.g., as an inset in Fig. 1(h)), to illustrate the correlation between blueshift magnitude and in-plane photon

momentum.

5. Phrases such as “Amazingly, in Fig. 4(c,g) we observe revival of the dyad coupling…” should be moderated. The observed revival follows naturally from the dispersion returning to a less pronounced detuning; it is interesting but not “amazing.”

6. In the RD SOC regime (≈1840 mV), while the Fourier-space locking is clear, the real-space interference pattern between the two spots is weak. The authors should comment on why interference is suppressed in real space—even when circularly polarized detection is applied (as mentioned in the SI, Fig. S4) the effect is quite small.

7. To complete the discussion, the paper should include an energy-resolved dispersion at 1880 mV similar to that shown for 0, 1600, and 1840 mV (Fig. 1). This would clarify why linearly polarized emission re-emerges in that regime.

In conclusion the work is interesting and potentially suitable for publication after substantial revision however I still have doubts about its novelty. The authors should really explicitly show what has been done and what is the added value of their work.

Reviewer #2

(Remarks to the Author)
This is an experimental study of weak-coupled microcavities containing a molecular dye and a liquid crystal. It is shown that a series of laser modes can be imprinted into the cavity plane that become coherently coupled via the lateral outflow of photons. It is then shown that by applying a voltage to the cavity that results in a change in the alignment of the liquid crystal, it is possible to change the optical properties of the cavity. In particular, by applying a voltage it is possible change the lasing emission properties of the cavity in Fourier space, in principle modifying the way that coupling between lasing nodes can be exploited. Finally, it is shown that by changing the relative pump polarisation of a chain of laser nodes, it is possible to change the coupling strength between nearest neighbours.

I think that this is a really interesting and comprehensive study that has been done very well. Here the novelty lies in the fact that the work is done in the weak coupling regime (rather than strong coupled) and that the optical properties of the cavity are tunable via electrical input. This introduces a new degree of control that will be of significant interest in advanced photonic and optoelectronic devices.

I have a few minor questions that the authors should consider as detailed below.

1. The authors claim the cavities are in the weak coupling regime. Is data available to show this?
2. How stable is the molecular dye in the cavities? How many laser pulses can be generated before degradation?
3. What is the cavity Q-factor and cavity photon lifetime? Is this lifetime consistent with the required outflow time / in-plane momentum that the authors claim is necessary to couple neighbouring laser nodes?
4. What is the largest separation that can be achieved between lasing while still achieving coherent coupling? What limits this time – photon leakage from the cavity?
5. Can the authors state specifically how the exchange of coherent photons drives the laser nodes into mutual coherence? Presumably photons from one laser trigger stimulated emission with a set phase and coherence? It would be good to have this explicitly stated. Indeed, do the authors have any idea of the timescale over which coupling occurs? It would be very interesting to measure this, however this is likely beyond the scope of the paper.

Reviewer #3

(Remarks to the Author)
This paper reports the realization of electrically controlled and optically reconfigurable lasing supermodes in an organic liquid crystal–filled microcavity operating in the weak light–matter coupling regime. The authors adapt the concept of polariton condensate arrays, previously demonstrated in GaAs-based microcavities to spatially separated lasing supermodes in the organic liquid-crystal microcavity. Remarkably, this paper reports phase-locked lasing behaviors within the Rashba–Dresselhaus spin–orbit coupling (RD SOC) regime, which is unattainable in conventional III–V semiconductor microcavities. Together with the authors’ previous works, this study highlights the originality of their unique platform. I appreciate the authors’ efforts in achieving such novel research. However, several important issues require clarification before the publication.

Major comments
1. Above the lasing threshold, the emission polarization follows that of the pump. However, under non-resonant excitation, the generated excitons are expected to lose the pump polarization rapidly. Could the authors clarify the mechanism responsible for this polarization behavior?

2. In Fig. 1(e), the lasing appears to occur at a finite in-plane momentum. Could the authors clarify why lasing takes place away from the lowest-energy photonic mode, which would typically be expected to provide the highest Q-factor and density of optical states?

3. The concept of the wavelength-dependent blueshift is somewhat ambiguous. Could the authors clarify what determines this dependence? Is it a quantitatively defined values that can be extracted from the data, or merely a phenomenological observation?

4. The blueshift becomes smaller under negative detuning. While this behavior is understandable in the strong-coupling regime due to the reduced excitonic fraction, its origin in the weak-coupling regime is not clear. Could the authors clarify how detuning affects the blueshift in this case?

5. The authors state that Frenkel excitons do not support repulsive interactions. In that case, what is the physical origin of the localized potential induced by the tightly focused pump? what is the “potential” in the weak-coupling regime? Could the authors elaborate on how such a potential is defined in a purely photonic lasing system without exciton–exciton interaction? How does this description account for the observed photon outflow from the pumped spot?

6. The authors repeatedly mention “room-temperature” and “weak coupling regime,” because they employ the concept of polariton studies. However, this seems to dilute rather than strengthen the novelty of the work. Conventional GaAs-based VCSELs already demonstrate high-quality lasing at room temperature. And since author’s LCMC system does not involve polariton lasing or cryogenic conditions, it is unclear why these aspects need to be highlighted. Could the authors better justify the necessity of emphasizing room-temperature and weak-coupling operation in this context?

Minor comments
7. The definition of the radius k_l is unclear. Please specify its meaning and indicate it in figures.

8. In Fig. 1(c–e), the dashed lines make the PL features difficult to see; adjusting their color or thickness would improve the data visibility.

9. The sentence “It has been shown [48, 49] that, in organic systems, the blueshift primarily arises from the saturation of molecular transitions and the associated refractive index change and hence, is present in both weak and strong light-matter coupling regimes and, importantly, is wavelength dependent, see Fig 1(c,d,e).” is difficult to follow due to its length and multiple clauses.

10. It is unclear whether the term “supermode” is newly introduced in this work or adopted from existing literature. If it is a new concept, please provide a clear definition. Otherwise, a proper reference should be cited when the term first appears.

11. Figure 3 appears somewhat disconnected from the main flow of the manuscript. It might improve readability if this figure is moved after Figure 6 or placed in the Supplementary Information. (This is just a suggestion.)

Reviewer #4

(Remarks to the Author)
I co-reviewed this manuscript with one of the reviewers who provided the listed reports. This is part of the Nature Communications initiative to facilitate training in peer review and to provide appropriate recognition for Early Career Researchers who co-review manuscripts.

Version 1:

Reviewer comments:

Reviewer #1

(Remarks to the Author)
The authors have satisfactorily addressed my comments, and the revised manuscript is acceptable in its present form.

Reviewer #3

(Remarks to the Author)
The authors have addressed all the concerns raised in the previous review. I have no further comments. Therefore, this manuscript is suitable for publication in its current form.

Reviewer #4

(Remarks to the Author)
I co-reviewed this manuscript with one of the reviewers who provided the listed reports. This is part of the Nature Communications initiative to facilitate training in peer review and to provide appropriate recognition for Early Career Researchers who co-review manuscripts.

Open Access This Peer Review File is licensed under a Creative Commons Attribution 4.0 International License, which permits use, sharing, adaptation, distribution and reproduction in any medium or format, as long as you give appropriate credit to the original author(s) and the source, provide a link to the Creative Commons license, and indicate if changes were made.
In cases where reviewers are anonymous, credit should be given to 'Anonymous Referee' and the source.
The images or other third party material in this Peer Review File are included in the article's Creative Commons license, unless indicated otherwise in a credit line to the material. If material is not included in the article's Creative Commons license and your intended use is not permitted by statutory regulation or exceeds the permitted use, you will need to obtain permission directly from the copyright holder.
To view a copy of this license, visit https://creativecommons.org/licenses/by/4.0/

Response to Reviewers' Comments

We sincerely thank the reviewers for their critical reading of our manuscript, their constructive comments, and their requests for clarification, which have strengthened and focused our work. In response to reviewers' comments, we provide a point-by-point answer to each comment and note the resulting changes to our manuscript.
We hope that the current version of the improved manuscript remains suitable for recommendation in Nature Communications.

Reviewer #1 (Remarks to the Author):

The manuscript by Dovzhenko et al. reports on the realization of spatially extended lasing "supermodes" in an organic liquid-crystal microcavity, operating in the weak coupling regime at room temperature. The authors demonstrate electrical control over the coupling between optically pumped lasing spots and the emergence of phase-locked extended emission patterns. Despite the blueshift of the emission stops once lasing locks to the cavity mode, which is the signature of lasing in the weak coupling regime, this is sufficient to enable a coherent outflow of photons that mediates coupling between different spatially separated lasing spots.

Overall, the experimental work is well done, and the topic is of potential interest to the community interested on reconfigurable photonic devices for which working at room temperature is essential. However, while the results are sound, the novelty compared to existing literature is not entirely clear and should be discussed more explicitly. In particular, similar concepts—both in inorganic (III–V, II–VI) systems and in recent room-temperature polaritonic platforms—have been reported. The authors should better position their work with respect to prior results. The demonstration of coupled lasing states and "supermodes" recalls several works in III–V polariton systems, and more recently also in room-temperature materials. For instance, Tao et al., Nat. Mater. 21, 761 (2022) [Ref. 24] already showed room-temperature polaritonic XY spin Hamiltonians with very nice data.

Likewise, the concept of electrically controlled polarization switching in hybrid or liquid-crystal cavities has been addressed by the same authors using the same system (see Ref. 41).

The manuscript currently lists these references under general discussions putting them here and there (e.g., "spin simulation"), but it should explicitly acknowledge what is new here beyond those achievements.

...

In conclusion the work is interesting and potentially suitable for publication after substantial revision however I still have doubts about its novelty. The authors should really explicitly show what has been done and what is the added value of their work.

Authors' reply: We thank the referee for appreciating our effort and highlighting the quality of the research outcomes. We agree that significant progress in all optical lattices has been made previously with exciton-polariton condensate systems, both in inorganic (III–V, II–VI) systems at cryogenic temperatures, and with perovskite-based microcavities Tao et al. [2022], Peng et al. [2024] at room temperature. However, it does not undermine the novelty of our work, which is manifold.

Firstly, we present the pioneering realization of ballistically coupled and optically reconfigurable arrays of coherent emitters in the weak coupling regime. Previous reports on phase-locked arrays of coherent emitters in planar microcavities were primarily realized either with ballistic polaritonic condensates (in-plane near field coupling) Alyatkin et al. [2021], Tao et al. [2022], or photonic lasing spots through sophisticated far-field feedback (coupling) Pal et al. [2020].

In contrast, our results were achieved by locally modifying the refractive index of the cavity through optical pumping, resulting in coherent photon outflow from individual localized lasing states. This is similar to ballistic polariton condensates but now entirely within the weak light-matter coupling regime, where excitonic interactions play a negligible role. Even though part of our results (Sec. 2.2) visually recalls previous studies of strongly-coupled systems, we emphasize the novel underlying physics of the in-plane phase-locking hereby observed.

Besides the novelty of demonstrating phase-locked extended lasing states in hybrid organic and liquid-crystal cavities with distinct tunable parameters, we also highlight the technological benefits of our approach. Despite the high quality of the results obtained in Tao et al. [2022] at room temperature with polaritonic condensates, we bring the first realization of coupled arrays of coherent emitters employing a microcavity based on traditional laser dye, which is possible due to the weak coupling operation regime. Additionally, this weak coupling regime is easier to realize technologically and offers greater flexibility due to its enhanced adaptability. Moreover, the use of laser dyes makes the microcavities more robust towards photobleaching and degradation, while offering easier fabrication and straightforward integration with birefringent liquid crystals (LC) inside the cavity.

The latter brings us to the **second** novelty of our study: the wide-range electrical tunability over the phase-locking mechanism between multiple lasing spots. The resulting extended lasing states in our sample can be simultaneously wavelength- and polarization-tuned. These particular tuning capabilities were indeed shown previously in a similar LC system for both photon Muszyński et al. [2022] and polariton Łempicka-Mirek et al. [2024] (Ref. 41) lasing regimes, albeit for the case of individual lasing state. The electrical tunability of the phase-locking mechanism between multiple individually pumped lasing spots demonstrated in current study is inherently novel effect though enabled by some properties of the system previously reported in Muszyński et al. [2022] and Łempicka-Mirek et al. [2024]. In previous studies the polarization of the emission from an individual lasing spot is shown to be electrically tuneable. Importantly, in current study we demonstrate continuous electrical tunability of the in-plane coupling parameter between the individually pumped lasing spots. We use the electrical tunability of the cavity modes as a tuning knob to control the value of the wavelength-dependent blueshift. The latter is shown to be responsible for creating the

outflow of coherent photons (non-zero in-plane momentum) in the cavity plane, which carries the phase-information to neighboring lasing states and establishes their mutual coherence. By electrically tuning the cavity modes and hence the corresponding blueshift and in-plane momentum emission from each lasing state, we can switch on and off the coupling between neighboring lasing states in a reversible manner. We emphasize that such electrical tuning of coupling between optical lattice sites was not previously realized in any strongly or weakly coupled system to the best of our knowledge.

The third novel aspect of our study comes from the highly birefringent nature of the LCs, which allows us to electrically tune the cavity dispersion into the so-called Rashba-Dresselhaus photonic spin-orbit coupling regime Rechcińska et al. [2019]. From this effect, we were able to realize directionally dependent spin-resolved coupling between spatially separated lasing spots, which is another unique property of our system that is harder to achieve in conventional inorganic semiconductor microcavities Dovzhenko et al. [2023].

The fourth major novelty of our study is the demonstration of going beyond the simple nearest-neighbour coupling picture between spatially separated lasing spots to next-nearest-neighbors, all at room temperature and in the weak coupling regime. This is important for the potential application of optical lattices as it offer opportunities to explore unconventional connectivity regimes in networks of coherently coupled arrays with non-planar graph topologies. Some experimental approaches have been successfully tested to overcome this constraint in traditional inorganic strong-coupled microcavities with polariton condensates Dovzhenko et al. [2023], Alyatkin et al. [2020] To the best of our knowledge, however, no such demonstration exists at room temperature, either in weak- or strong-coupling regimes. We achieve this by exploiting the polarization transfer from the pump spots to the gain medium inherited by the lasing spots. The precise dispersion engineering through the LC permits us to minimize cavity polarization anisotropy (birefringence) while subsequently taking advantage of the gain-induced polarization anisotropy in the organic dye to create lasing spots at the same energy but opposite polarization. As a result, we can controllably switch off the coupling between the nearest neighbors, while next-nearest-neighbors are still strongly interacting.

We can summarize the four major aspects of novelty in our study as follows: the first realization of ballistically coupled and optically reconfigurable arrays of coherent emitters in the weak coupling regime, unique electrical tunability of in-plane coupling, directional dependent spin-resolved coupling between spatially separated lasing states in Rashba-Dresselhaus spin-orbit coupling regime, and first demonstration of next-nearest-neighbor coupling regime between the photonic lasing spots and at room temperature.

Hence, we introduce a new physical platform and approach towards optical lattice physics, which is both based on new physical phenomena and capable of entering otherwise unattainable regimes of operation, offering significant technological benefits over existing solutions. We want to separately emphasize that, to the best of our knowledge, neither of those four novelty aspects were previously demonstrated in inorganic polaritonic (III–V, II–VI) systems at cryogenic temperatures or in perovskite-based microcavities Tao et al. [2022], Peng et al. [2024] at room temperature. In regard to the novelty of our work in relation to the previously published studies using LC microcavities Łempicka-Mirek et al. [2024], Muszyński

et al. [2022] [Ref. 41 and 42] we want to note the following. Firstly, all the previous studies concerned only individual lasing states and no in-plane phase-locking was demonstrated unlike in the current study. Secondly, the electrical tuning capabilities reported previously concerned **tuning of wavelength and polarization** of a single laser, while in current study we present the **electrical tunability of the in-plane phase-locking mechanism** between multiple lasing spots (see **second** novelty aspect for the details above).

We agree with the reviewer that we need to highlight those aspects of novelty in our study more explicitly and introduced the following changes in the manuscript introduction and discussion sections:

Ballistic in-plane coupling between coherent emitters at room temperature was though demonstrated with perovskite-based microcavities, where two-dimensional condensate lattices were realized for simulation of XY Hamiltonian Tao et al. [2022], Peng et al. [2024]. *It is important to note that this approach requires demanding technology to grow large and uniform single-crystal perovskites inside the microcavity, which needs to be simultaneously robust towards photobleaching for continuous operation, limiting the applicability*

In contrast, weakly-coupled organic microcavities offer application-friendly, well-developed, and relatively easy fabrication technology, robustness of operation, and wide-range electrical tuning capabilities enabled by the integration with highly birefringent liquid crystals Muszyński et al. [2022]

We note that previously reported electrical tuning capabilities in similar LCMC systems Łempicka-Mirek et al. [2024], Muszyński et al. [2022] *concerned the emission properties of an individual lasing state or polariton condensate. The wavelength tuning arose from the cavity-mode renormalization induced by the rotation of highly-birefringent LCs under an external electric field, while the polarization and condensation threshold tuning originated from the realization of the RD SOC regime. In contrast, in the present study we present the electrical tunability of the in-plane phase-locking mechanism between multiple lasing spots. While the system remains above the lasing threshold in all operation regimes, the in-plane phase-locking between the separately pumped lasing spots is controlled electrically by modifying the Fourier-plane emission distribution of each lasing state. This unique functionality is enabled by the combined effect of electrically tunable cavity modes and the wavelength-dependent blueshift of the coherent emission forming a highly localized optically-induced potential.*

However, in the future larger two-dimensional lattices of coupled lasing states can be developed with the prospect of exploring non-Hermitian physics, topologically protected states Tsesses et al. [2018], Yang et al. [2022] *, and as a robust platform for photonic simulator* Mahler et al. [2024], Parto et al. [2020], Tao et al. [2022] *and all-optical neural networks* Zuo et al. [2019], Chen et al. [2023], Matuszewski et al. [2024] *operating in a weakly coupled regime at room temperature and offering the benefits of electrical tuning of the wavelength, polarization, and coherence as well as non-trivial coupling capabilities between the lattice*

nodes

Comment #1 (R1C1):

More specific comments:

1. The term supermode is used even when a single spot is lasing. The authors should clarify what they mean by "supermode". Conventionally, a supermode implies coherent coupling between at least two sub-modes; in which sense this is valid here?

Authors' reply: We thank the referee for drawing our attention to this point. In our paper, we define the lasing supermode as a spatially extended coherent lasing state consisting of two (dyad supermode), three (triad supermode), or more phase-locked individually pumped lasing spots, which are coupled via in-plane coherent photon exchange. We agree with the statement that the term "supermode" should be used to address a coherently coupled state between at least two lasing spots. For this reason, we exclusively use it for lasing "dyad" (Sec. 2.2, 2.3, and schematic in Fig.1), "triad" (Sec. 2.4), and the two-by-two lattice case, while the single-spot excitation case is addressed as a "single lasing state".

Following the reviewer's suggestion, we decided to add a clarifying sentence earlier in the manuscript that defines the use of "supermode" terminology:

The term "supermode" [Paoli et al. [1984], Mehuys et al. [1988], Ginis et al. [2023]] is used here to refer to the spatially extended coherent lasing state consisting of two or more phase-locked spatially separated and individually pumped lasing spots, which are coupled via in-plane coherent photon exchange.

Comment #2 (R1C2): *2. Figures 3 and 4 appear to be referred to in reverse order in the text. Please check the sequence and ensure consistent references throughout the manuscript.*

Authors' reply: Currently, Fig. 3 is first referred to on page 8, last paragraph of Sec. 2.2, and Figure 3 itself appears at the top of page 9. While Figure 4 is first referred to in the second paragraph of Sec. 2.3, and Figure 4 itself is shown at the top of page 10. Thus, we consider the Figure referencing to be consistent. However, if the issue persists, we would be happy if the reviewer could direct us to the exact location where the referencing requires correction.

Comment #3 (R1C3): *3. The role of the pump polarization is not fully clear. The authors state that "the polarization of the lasing state is inherited from the pump polarization," but the presented data seem instead to indicate that the emission polarization follows the cavity eigenmode that lases at threshold. For instance, in Fig. 1(f), the emission appears vertically*

polarized (parallel to the lower-energy cavity mode). Later, for three-spot configurations, the emission polarization can be tuned by changing the pump polarization. Does this tuning also shift the emission energy? The authors should clarify whether the lasing state polarization is dictated by the cavity anisotropy or by pump-induced gain anisotropy, and discuss whether both effects compete.

Authors' reply: We thank the referee for the question and the possibility to further elaborate on the matter. Regarding the data presented in Fig.1(g) (previously Fig.1(f)), the emission is vertically polarized for 0 mV and 1600 mV voltage due to the vertical polarization of the pump used in this experimental section. The possibility of switching the linear polarization of the emission above the threshold is shown in details in Supplemental Information Sec. S1.1, where we tune the polarization of the pump for the case of 0 mV voltage, corresponding to only minor (at the order of magnitude of the emission linewidth) energy difference between the H (horizontal) and V (vertical) cavity modes. The same regime is also used in Sec 2.4 for the three-spot configuration. As such, we consider that in this regime (at 0 mV external voltage applied), the tuning of the pump and emission polarization doesn't lead to a significant change in the energy. However, we agree with the reviewer that, generally speaking, both effects (the cavity anisotropy and pump-induced gain anisotropy) can play a role and compete. Indeed, the cavity anisotropy can be strong enough to favor a particular polarization profile of the emission, such that the emitted light can be polarized perpendicular to the pump. In the experiments presented in this study, we purposely avoided operating in this regime.

To provide a better understanding for readers and avoid confusion, we added the following sentences to the Supplemental Information, Sec 1.1, describing the individual lasing state emission properties.

Here we excited an individual lasing state using a linearly polarized pump in the regime of small TE-TM splitting near $k_\parallel = 0$, where the difference in energy of the emission above the lasing threshold for horizontal and vertical polarizations remains negligible, and gain-induced anisotropy defines the polarization of the lasing state, inherited from the pump. Notably, apart from the gain-induced anisotropy allowing for the control of the emission polarization above the threshold, cavity anisotropy can be introduced as a competing mechanism under specific detuning conditions. In the case of a large relative spectral detuning between the horizontal and vertical cavity modes, the gain difference can become strong enough to favor a particular polarization of the emission such that the linear polarization of the emission above the threshold can be perpendicular to the polarization of the pump. In the experiments presented in the current study, we avoided such a regime of operation.

We also decided to add the following sentences to Sec 2.1 of the main text, describing the polarization behavior.

Despite the linear polarization of the laser pump, the intermolecular energy transfer en-

ables the fast depolarization of the excited states and unpolarized emission below the lasing threshold Chang et al. [2007], Musser et al. [2017], Yagafarov et al. [2020], Plumhof et al. [2014] for the case of relatively small H-V splitting near $k_{\parallel} = 0$ comparing to the broadening of the organic dye emission.

The transition from unpolarized emission below the lasing threshold to highly polarized emission in the lasing regime is due to the carrier recombination, enabled by the offset of stimulated emission, happening faster than the depolarization effects in the dye.

Comment #4 (R1C4): *4. It would be helpful to explicitly show the small blueshift residual for the 1600 mV case, where no ballistic expansion is observed (e.g., as an inset in Fig. 1(h)), to illustrate the correlation between blueshift magnitude and in-plane photon momentum.*

Authors' reply: We thank the referee for the suggestion, and we added the corresponding information to Fig.1(i) (previously Fig.1(h)).

Comment #5 (R1C5): *5. Phrases such as "Amazingly, in Fig. 4(c,g) we observe revival of the dyad coupling..." should be moderated. The observed revival follows naturally from the dispersion returning to a less pronounced detuning; it is interesting but not "amazing."*

Authors' reply: We accept that the use of such a word is stylistically questionable. However, our goal was to emphasize that the coupling revival occurred in a significantly different regime of operation. Since the horizontally and vertically polarized modes are again in resonance at 1840 mV, while being of different parity, the Rashba-Dresselhaus spin-orbit coupling regime is realized, in which the emission is present with different polarization (circularly polarized) and re-distributed in Fourier space. As such, we consider that the regime is significantly different from the situation at 0 mV, despite the similar values of blueshift observed, and find the revival of the coupling to be quite "amazing". However, we agree that its origin is understandable, and it can be numerically reproduced within our theoretical model.

To converge the text to a more classical wording, we replaced the word "amazingly" with "interestingly" when addressing the revival of phase-locking.

Comment #6 (R1C6): *6. In the RD SOC regime (≈ 1840 mV), while the Fourier-space locking is clear, the real-space interference pattern between the two spots is weak. The authors should comment on why interference is suppressed in real space—even when circularly polarized detection is applied (as mentioned in the SI, Fig. S4) the effect is quite small.*

Authors' reply: We thank the reviewer for drawing attention to this question. Indeed, the experimentally observed effect of fringes appearing between the lasing spots is reduced even after detecting opposite components of circularly polarized light separately. We attribute this effect to the real- and Fourier-space redistribution of the emission from each of the coupled lasing spots in the presence of spin-orbit coupling. In the case of the Rashba-Dresselhaus regime, two circular components are separated in Fourier space, meaning that we have spin-dependent directional coupling, e.g., σ^+ emission propagates to the right, while σ^- propagates to the left, as shown in Figure 4 of the main manuscript and Figure 4 of Supplemental Information. Hence, instead of two counter-propagating waves of the same polarization, interfering constructively or destructively to form a pronounced pattern, we have two phase-shifted waves of opposite polarization, propagating in opposite directions which do not interfere directly between the lasing spots but do provide polarization dependent directional phase-injection (σ^+ propagates from left lasing spot to right and σ^- vice versa as follows from their respective distribution in Fourier space, with only a small fraction of intensity going in opposite direction). We note that the same effect of limited fringes visibility and low total intensity between the lasing spots at 1840 mV is clearly present in numerical simulations (Fig. 5 of the main manuscript). Once the polarization-resolved Fourier-space distribution of the emission from each lasing state becomes symmetric again relative to the k_y axis (1880 mV voltage case in Fig.5(c)) the interference fringes between the lasing spots in the real space are gradually restored. We also want to emphasize that the revival of phase-locking between the lasing spots in the RD SOC regime is further confirmed by the corresponding revival of coherence in the spatially resolved interferometry measurements performed in real space (see Sec 1.2 in the SI).

To help readers better understand this phenomenon, we introduced the corresponding discussion in Supplemental Information, Sec. S1.3 and made clarifying changes to the text in the Sec. 2.3 of the main manuscript.

We state in Sec. 2.3 of the main manuscript that we observed the revival of the coupling between separated lasing states in a dyad configuration when the system enters the RD SOC regime. However, while the signature fringe pattern is clearly observed in Fourier-space and further evidenced by the coherence revival in interferometry data (SI Sec. S1.2), its visibility is lowered in the real-space image of total emission. We attribute this effect to two contributing factors stemming from the change of the Fourier space emission pattern in the presence of spin-orbit coupling. Firstly, due to the spin-dependent redistribution of the emission in Fourier space, instead of two counter-propagating waves of the same polarization, interfering constructively or destructively to form a pronounced pattern, two phase-shifted waves of opposite polarization are present, which do not interfere directly. The consecutive spatial shift between the emission patterns of opposite circular polarisation makes fringe pattern in the real-space total intensity distribution (Fig.4(c) in the main text) drastically reduced. In Fig.S4 we show the corresponding experimentally measured polarisation-resolved real-space distribution of the emission from a coupled dyad state in the RD SOC regime at an 1840 mV external voltage. Characteristic fringe pattern is observed for both polarisation components.

We note that the visibility of the fringes in polarisation-resolved images remains lower than that typically observed for a linearly polarized dyad, which can be attributed to the second consequence of the RD SOC regime. Both circular polarization components have a dominant direction of propagation with σ^+ polarised emission propagating from the left lasing spot to the right and σ^- polarised emission vice versa with only a small fraction of the intensity propagating in the opposite direction [as follows from their respective distributions in Fourier space]. As a result, while phase-locking is still achieved by directional propagation of the emission components between the lasing spots, each of the polarisation components between the lasing spots does not have a counter-propagating wave of similar intensity and polarisation to form a strong interference pattern with. The same effect of limited fringes visibility and low total intensity between the lasing spots at 1840 mV is clearly present in numerical simulations (Fig. 5 of the main manuscript). Once the polarization-resolved Fourier-space distribution of the emission from each lasing state becomes symmetric again relative to the k_y axis (1880 mV voltage case in Fig.5(c) in the main text) the interference fringes between the lasing spots in the real space distribution of the total emission are gradually restored.

We attribute this to the real- and Fourier-space redistribution of the phase-locked dyad emission in the presence of spin-orbit coupling when each of the circularly polarized components propagates in opposite directions and has an oscillating intensity pattern of lower intensity, spatially shifted relative to each other.

We note that the experimentally observed effect of reduced interference fringes visibility in the RD SOC regime, accompanied by the clear phase-locking in Fourier space, is similarly observed in numerical simulations.

Comment #7 (R1C7): *7. To complete the discussion, the paper should include an energy-resolved dispersion at 1880 mV similar to that shown for 0, 1600, and 1840 mV (Fig. 1). This would clarify why linearly polarized emission re-emerges in that regime.*

Authors' reply We thank the reviewer for the suggestion. Energy-resolved dispersion at 1880 mV was shown in SI of the original version of the manuscript. Following the recommendation from the reviewer, we added the data, corresponding to the 1880 mV regime, to Fig.1 and introduced the subsections in the Figure to improve clarity.

Reviewer #2 (Remarks to the Author):

This is an experimental study of weak-coupled microcavities containing a molecular dye and a liquid crystal. It is shown that a series of laser modes can be imprinted into the cavity plane that become coherently coupled via the lateral outflow of photons. It is then shown that by applying a voltage to the cavity that results in a change in the alignment of the liquid crystal, it is possible to change the optical properties of the cavity. In particular, by applying a voltage it is possible change the lasing emission properties of the cavity in Fourier space, in principle modifying the way that coupling between lasing nodes can be exploited. Finally, it is shown that by changing the

relative pump polarisation of a chain of laser nodes, it is possible to change the coupling strength between nearest neighbours.
I think that this is a really interesting and comprehensive study that has been done very well. Here the novelty lies in the fact that the work is done in the weak coupling regime (rather than strong coupled) and that the optical properties of the cavity are tunable via electrical input. This introduces a new degree of control that will be of significant interest in advanced photonic and optoelectronic devices.

I have a few minor questions that the authors should consider as detailed below.

Comment #1 (R2C1): *1. The authors claim the cavities are in the weak coupling regime. Is data available to show this?*

Authors' reply: We thank the referee for the opportunity to highlight one of the key novelty aspects of the study: the observed in-plane phase-locking of spatially separated lasing states is achieved in the weak coupling regime. Indeed, it needs to be proved robustly that the microcavity was not driven to the strong coupling regime. Firstly, we note that we have not observed any anticrossing features while tuning the sample both electrically and by displacing the excitation position due to the wedged type of the microcavity, which would have otherwise been a signature of the strong coupling. Furthermore, as the emission from the upper polariton branch is known to be hard to observe Neuman and Aizpurua [2018] in certain systems, we implemented the quantitative analysis to confirm the weak coupling regime of operation. It is based on the extraction of the effective mode mass by fitting the set of electrically detuned cavity modes with a parabolic function over the wide range of detuning values, which is presented in the Supplementary Information, Sec. S2. We observed that the effective mass remained approximately constant, which supports the claim of the purely photonic nature of the cavity modes observed and hence, the weak coupling regime of operation.

To highlight the data showing the weak coupling regime, we added the following sentence to the manuscript, which signposts the reader towards relevant data in the Supplementary Information.

Detailed analysis supporting the claim that the LCMC is operating in the weak light-matter coupling regime (as opposed to the strong coupling regime) is provided in Supplementary Information, Sec.S2

Comment #2 (R2C2): *2. How stable is the molecular dye in the cavities? How many laser pulses can be generated before degradation?*

Authors' reply: We thank the reviewer for the possibility to address this question. Under the experimental conditions used in the manuscript to obtain phase-locked arrays of lasing states, the molecular dye proved to be stable. Despite the reported results being measured in a single-shot regime, we also tested the multi-shot regime of excitation, with a 20 Hz repetition rate and a 5 ns pulse length. While we have not done extensive analysis, which was at the moment out of the scope of the paper, we can confirm that no apparent degradation of the lasing was observed for more than 10 minutes of excitation, yielding more than 12,000 laser pulses generated. We believe that the stability of the supermode lasing regime and its dependence on the excitation parameters is an interesting research direction, and can be explored in a separate study.

We added the following sentence in the Supplementary Information to comment on the photostability of the lasing.

Photostability of the emission was tested in the multi-shot regime of the excitation, with a 20 Hz repetition rate and a 5 ns pulse length. No apparent degradation of the lasing was observed for more than 10 minutes of excitation, yielding more than 12,000 laser pulses generated.

Comment #3 (R2C3): *3. What is the cavity Q-factor and cavity photon lifetime? Is this lifetime consistent with the required outflow time / in-plane momentum that the authors claim is necessary to couple neighbouring laser nodes?*

Authors' reply: We thank the referee for this detailed question regarding the nature of the investigated system. The Q-factor of the microcavity is around 300, which corresponds to the single photon cavity lifetime of 91 fs. It should be noted that above the lasing threshold, the linewidth narrows significantly due to increased coherence in the system. The coherence lifetime in a lasing state then exceeds the lifetime of a single photon in the cavity, and was measured using Mach-Zehnder interferometer to be around 8.5 ps (see in the Figure below). This allows propagation over distances up to ~ 100 μm considering the in-plane momentum ~ 1 μm^{-1}. The experimentally observed maximum separation distance in a dyad supermode can be decreased due to the non-homogeneity of the molecule distribution or other imperfections in the cavity. In the investigated sample, coupling of neighbouring laser nodes was experimentally observed mostly at distances up to 20 μm with larger coupling distances at specific sample locations favoring coherent photon propagation, which is within the theoretically estimated limit. We consider temporal and spatial coherence properties and phase-propagation in the arrays of phase-locked lasing states in our liquid-crystal microcavity systems to be a direction of further research and a separate publication, as referee also mentions in comment R2C5.

Comment #4 (R2C4): *4. What is the largest separation that can be achieved between*

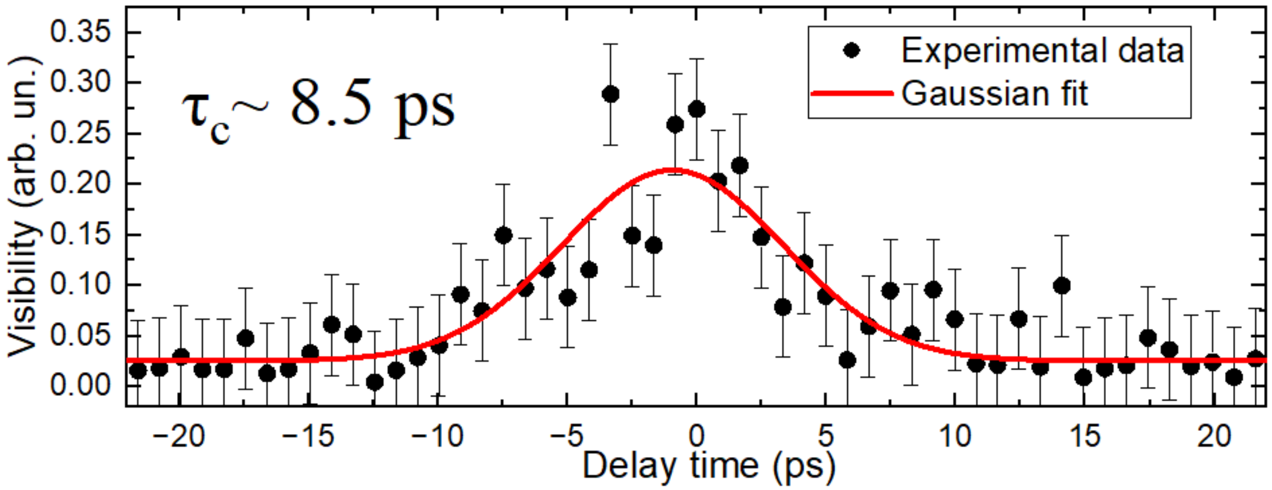

Figure L1: **Temporal coherence of the lasing state**. Visibility of the interference fringes dependence on the time delay between the two arms of the Mach-Zehnder interferometer.

lasing while still achieving coherent coupling? What limits this time – photon leakage from the cavity?

Authors' reply: We thank the referee for the question, which is a follow-up question to the previous comment (R2C3). The maximum distance, where phase-locking is demonstrated in this study, is position-dependent due to some level of inhomogeneity and equals $\approx 20\mu$m for the majority of the cavity surface and up to to $\approx 30\mu$m in the regions favoring better coherent photon outflow, while the theoretical limit is calculated to be ~ 100 μm. We attribute the latter limitation to the lasing state coherence lifetime. As explained in our reply to comment R2C3, local conditions in the sample resulting from inhomogeneity can decrease the experimentally achievable coupling distances.

Comment #5 (R2C5): *Can the authors state specifically how the exchange of coherent photons drives the laser nodes into mutual coherence? Presumably photons from one laser trigger stimulated emission with a set phase and coherence? It would be good to have this explicitly stated. Indeed, do the authors have any idea of the timescale over which coupling occurs? It would be very interesting to measure this, however this is likely beyond the scope of the paper.*

Authors' reply: We thank the referee for the possibility to further elaborate on the mechanism of lasing state coupling. As a result of molecular transition saturation, a local change in the refractive index occurs [Yagafarov et al. [2020], Putintsev et al. [2023]]. This change causes photons to ballistically flow from the pump spot radially in all directions in the cavity plane including the direction towards neighbouring lasing state, which is parallel to the interaction axis between two neighboring nodes [Kippenberg et al. [2002]]. Outflowing pho-

tons reaching the neighbouring energy-degenerate source induce stimulated emission with a phase seeded by another node. The coherence of the cavity mode lifetime above the lasing threshold, approximately 8.5 ps, allows theoretically propagation up to a 100 micrometres [up to 30 micrometres observed experimentally]. In this way, the nodes lock the phase and share coherence. The phenomenon is closely related to a previous study by one of the authors where a classical coupled-oscillator model with time delay was able to describe similar synchronization between ballistic sources of polaritons [Töpfer et al. [2020]].

To help readers better understand the phenomenon under study and avoid confusion, we altered the following sentence:

Fig.2(a-f) shows the experimentally measured real- (a-c) and momentum- (d-f) space distributions of the PL from the spatially extended supermode lasing state formed due to the mutual exchange of coherent photons out-flowing from each of the two individually pumped lasing spots and establishing mutual coherence between them via the stimulated emission process. Once the coupling between the lasing spots is established, they act as coherently coupled oscillators, which relative phase is defined by the distance between them.

Reviewer #3 (Remarks to the Author):

This paper reports the realization of electrically controlled and optically reconfigurable lasing supermodes in an organic liquid crystal–filled microcavity operating in the weak light–matter coupling regime. The authors adapt the concept of polariton condensate arrays, previously demonstrated in GaAs-based microcavities to spatially separated lasing supermodes in the organic liquid-crystal microcavity. Remarkably, this paper reports phase-locked lasing behaviors within the Rashba–Dresselhaus spin–orbit coupling (RD SOC) regime, which is unattainable in conventional III–V semiconductor microcavities. Together with the authors' previous works, this study highlights the originality of their unique platform. I appreciate the authors' efforts in achieving such novel research. However, several important issues require clarification before the publication.

Major comments:

Comment #1 (R3C1): *1. Above the lasing threshold, the emission polarization follows that of the pump. However, under non-resonant excitation, the generated excitons are expected to lose the pump polarization rapidly. Could the authors clarify the mechanism responsible for this polarization behavior?*

Authors' reply: We thank the referee for the question and the possibility to further elaborate on the matter. Unlike the excitation in inorganic materials, the emission polarisation from organic dye molecules has a linear basis, being defined by the molecular dipole moment and hence, the relative spatial orientation of each molecule. Since the organic dye molecules are randomly oriented in the ensemble inside the microcavity at zero voltage, all possible dipole orientations are available, and when a non-resonant linearly polarized excitation is

projected on the sample, it initially excites only a subset of molecules with a specific molecular dipole orientation, corresponding to the polarization of the pump. Then, fast mechanisms of depolarization come into place. The intermolecular energy transfer is considered the major process responsible for the depolarization of fluorescence, even at relatively low concentrations of the dye molecules Chang et al. [2007], Musser et al. [2017]. Hence, below the lasing threshold, the emission polarization doesn't follow the polarization of the pump and remains unpolarized as long as the H-V splitting of the cavity modes near $k_{\parallel} = 0$ remains small relative to the broadening of the organic dye emission. However, above threshold, the emission becomes stimulated, and the drastically shortened relaxation is faster than the effects of depolarization. Hence, the emission polarization above the lasing threshold follows that of the pump under the assumption of similar gain values of the media for TE and TM modes. We also note that this effect is accounted for in our numerical simulations (Sec. 4.3) as a γ_{XX} term in the equation describing the dynamics of the excitonic reservoir with density $n_{H,V}$.

To help readers better understand the phenomenon under study and avoid confusion, we added the following sentences to Sec 2.1 of the main text to describe the polarization behavior.

Despite the linear polarization of the laser pump, the intermolecular energy transfer enables the fast depolarization of the excited states and unpolarized emission below the lasing threshold Chang et al. [2007], Musser et al. [2017], Yagafarov et al. [2020], Plumhof et al. [2014] for the case of relatively small H-V splitting near $k_{\parallel} = 0$ *comparing to the broadening of the organic dye emission.*

The transition from unpolarized emission below the lasing threshold to highly polarized emission in the lasing regime is due to the carrier recombination, enabled by the offset of stimulated emission, happening faster than the depolarization effects in the dye. Yagafarov et al. [2020], Plumhof et al. [2014].

Comment #2 (R3C2): *2. In Fig. 1(e), the lasing appears to occur at a finite in-plane momentum. Could the authors clarify why lasing takes place away from the lowest-energy photonic mode, which would typically be expected to provide the highest Q-factor and density of optical states?*

Authors' reply: We first want to draw attention to the property of the system that establishes emission at finite in-plane momentum not only in Fig.1(e) but also in Fig.1(c) and Fig.1(f), while in Fig.1(d) the emission occurs at the bottom of the dispersion band, similar to what is typically expected from a microcavity lasing system. In Fig.1(c), we observe a ring of intensity in Fourier space (of radius k_l), which characterizes the in-plane momentum of the coherent photon outflow. The Fourier image in Fig.1(e) has two rings, each of radius k_l, shifted symmetrically along the k_x due to the Rashba-Dresselhaus spin-orbit coupling.

Due to this unique property of the system, we were able to achieve in-plane propagation and phase-locking (even in the regime of 0 V external voltage) and also have an electrical tuning knob to control the coupling reversibly. In Fig.1(f) we show how the system is electrically tuned back to linear polarization operation regime through another anisotropic emission regime, where $\langle|k_y|\rangle > \langle|k_x|\rangle$.
The reason why lasing takes place at a higher energy than the bottom of the dispersion band in Fig.1(c,e,f) is due to the localized cavity photon blueshift induced by the tightly focused intense excitation spot. It has been shown previously that, for similar organic systems, the observed blueshift of the emission (whether in strongly or weakly coupled systems) is due to the saturation of molecular optical transitions, which changes the intracavity refractive index due to the optical excitation of the organic molecules Yagafarov et al. [2020]. Due to the spatially shaped excitation profile, the dielectric constant becomes non-uniform across the microcavity plane. The refractive index change in the pump region leads to a renormalization effect of the cavity energy modes and a corresponding blueshift of the emission. We treat such a distribution of the dielectric constant as an effective "potential" for photons with amplitude $\hbar G(P_H + P_V)$ in Eq. (6) in the main text. [We further discuss the origin of this blueshift and potential in our reply to Comments 3 and 5, correspondingly.]
Typically, the size (width) of the excitation spots used in studies on similar organic microcavities is on the order of tens of μm. This means they have a lower threshold for a given power density, which leads to a smaller effective potential gradient for the majority of the generated photons, even though the blueshift across the pump region is present Yagafarov et al. [2020], Plumhof et al. [2014], Li et al. [2022].
Here, we focused tightly the excitation of the pump spots to induce a sharp localized blueshift of the cavity modes, surrounded by the area with unchanged mode energy. Hence, a potential gradient is experienced by the photons that are dispersed from the center of the lasing spot, converting potential energy into kinetic energy and forming a coherent radial outflow away from the pumping region. [It is reminiscent of the effects observed in polaritonic condensates in GaAs microcavities, where the polariton interaction with the reservoir creates a local blueshift and a similar outflow of polaritons with non-zero in-plane momentum depending on the value of the blueshift is observed Töpfer et al. [2020].] Hence, coherent outflow photons obtain finite in-plane momentum, observed as a ring emission in the Fourier space of radius defined by the amount of pump-induced blueshift relative to the non-excited plane. In case of Rashba-Dresselhaus regime in Fig.1(e), the maximum in-plane momentum is further enhanced in k_x direction by the shift of the bottom of each of the paraboloid dispersions along the k_x direction.

To convey our message more clearly, in the manuscript we added the following sentences:

It can be seen in Fig.1(c,d,e,f), that the blueshift is present when the vertically polarized cavity mode energy at $k_x = 0$ is around 2.16 eV or higher. Such behaviour is expected assuming the proposed above blueshift mechanism associated with the saturation of the optical transitions. While the P580 dye absorption flattens around 0 below 2.15 eV, strong increase is observed above the 2.16 eV Mowatt et al. [2010], which, upon the saturation of the optical

transition, leads to the corresponding decrease of the refractive index and cavity mode renormalization under the optical pumping area.

In case of tightly focused (≈ 3 µm) optical pumping, it creates sharp gradient of the cavity mode energy, which acts as a localized potential sufficient to induce the outflow of coherent photons with finite average absolute value of in-plane momentum ($k_{||} \neq 0$) radially propagating away from the pumping spot in the cavity plane.

Comment #3 (R3C3): *3. The concept of the wavelength-dependent blueshift is somewhat ambiguous. Could the authors clarify what determines this dependence? Is it a quantitatively defined values that can be extracted from the data, or merely a phenomenological observation?*

Authors' reply: We thank the reviewer for the possibility to address this issue. By the wavelength-dependent blueshift we meant the dependence of the observed value of the emission blueshift above the lasing threshold on the wavelength of the bottom of the corresponding cavity mode [note that the wavelength of the non-resonant pump remains fixed in all of the experiments]. This dependence is dictated by the origin of the blueshift and the unique property of the system allowing for the electrical tuning of the cavity modes. We argued above that, contrary to classical III-V polaritonic systems, the origin of the blueshift observed in our organic microcavity is the saturation of the optical transition, corresponding to the change of refractive index and local renormalization of the cavity modes energy Yagafarov et al. [2020]. We emphasize that even though the dye used in the current work is not exactly the same as in Yagafarov et al. [2020], their chemical composition and optical properties are very similar, allowing us to consider that their behaviour in a similarly built microcavity should not drastically differ. The value of such blueshift depends on the relative energy between the bottom of the cavity photon dispersion band and the resonance energy of the optical transition in the intracavity media, which, in our case, is the absorption of P580 dye in liquid crystal solution Mowatt et al. [2010]. The wavelength dependence of the observed blueshift above the lasing threshold is determined by the value of cavity mode renormalization (relative to the unexcited cavity modes) due to the change of refractive index induced by the saturation of optical transitions. Since the numerical estimation of this effect would require extremely precise measurements of local organic dye molecules concentration, spatial distribution and optical accessibility, we quantitatively define the values of the blueshift by measuring the pump power dependence of the energy resolved emission in Fourier space for the individual lasing state and extract the energy of the maximum emission intensity around $k_x = 0$, Fig.1(i). To account for this effect in our numerical simulations (Sec. 4.3 of the main text), we introduce a phenomenological parameter G, chosen to fit the experimental measurements.

To help readers better understand the phenomenon of the wavelength-dependent blueshift, we decided to introduce the following discussion in the manuscript:

Indeed, it has been shown in Yagafarov et al. [2020] that the microcavity modes renormalization upon optical excitation can be described using the general Kramers-Kronig analysis of the organic dye complex refractive index. Assuming the decrease of the imaginary part of the refractive index due to the saturation of optical transitions, the corresponding change of the real part follows in the area of anomalous dispersion. Therefore, the blueshift value depends on the relative energy between the bottom of the cavity photon dispersion band and the resonance energy of the optical transition in the intracavity media, which, in our case, is the absorption of P580 dye in liquid crystal solution Mowatt et al. [2010]. It can be seen in Fig.1(c-f) that the blueshift is present when the vertically polarized cavity mode energy at $k_x = 0$ *is around 2.16 eV or higher. Such behaviour is expected assuming the proposed above blueshift mechanism associated with the saturation of the optical transitions, as the P580 dye absorption flattens around 0 below 2.15 eV, while experiencing a strong increase above 2.16 eV Mowatt et al. [2010] (see table in the inset Fig.1(i)), which, upon the saturation of the optical transition, leads to the corresponding decrease of the refractive index and cavity mode renormalization under the optical pumping area.*

Comment #4 (R3C4): *4. The blueshift becomes smaller under negative detuning. While this behavior is understandable in the strong-coupling regime due to the reduced excitonic fraction, its origin in the weak-coupling regime is not clear. Could the authors clarify how detuning affects the blueshift in this case?*

Authors' reply: We thank the reviewer for bringing attention to this important difference between our system and the ones in the strongly-coupled regime. Indeed, as the lasing states are purely photonic, the detuning doesn't affect the composition of coherent particles and doesn't change the effective mass (see Supplemental Information, Sec. S2). We highlight that, in Yagafarov et al. [2020], the authors also emphasize that the demonstrated optically induced nature of the blueshift in organic microcavities is not limited to strongly-coupled systems and can be observed in weakly coupled microcavities. As such, the blueshift itself can not be considered a signature of polaritonic condensation. In our case, the blueshift we observe above the lasing threshold experiences a dependence on the detuning of the cavity mode, which is achieved by applying external voltage. The physical origin of such detuning is described in detail in reply to previous Comment #3. In summary, the reason for the observed reduction in the blueshift value is the spectral detuning of the cavity mode relative to the resonance energy of the optical transition in the P580 dye, which can be estimated from the corresponding dye absorption spectra [Mowatt et al. [2010]]. Indeed, the absorption of the dye flattens around 0 below 2.15 eV, while experiencing a strong increase above 2.16 eV. We observe that this behaviour is translated to the corresponding drop in the blueshift when the cavity mode is detuned below 2.15 eV. This is in accordance with the assumption of the saturation of the optical transition being responsible for the blueshift observed. As no transition is present at this energy, the corresponding refractive index value doesn't change following local non-resonant excitation, and the lasing occurs at the bottom of the dispersion

curve. We note that the refractive index still changes at higher energies (above 2.15 eV), but it doesn't affect negatively detuned cavity modes.

To help readers better understand the phenomenon under study and avoid confusion, we decided to extend the text that we added to address the previous Comment #3 to clarify both the blueshift mechanism and the particular regime of detuning below 2.15 eV. For completeness, below we write the updated text below:

Indeed, it has been shown in Yagafarov et al. [2020] that the microcavity modes renormalization upon optical excitation can be described using the general Kramers-Kronig analysis of the organic dye complex refractive index. Assuming the decrease of the imaginary part of the refractive index due to the saturation of optical transitions, the corresponding change of the real part follows in the area of anomalous dispersion. Therefore, the blueshift value depends on the relative energy between the bottom of the cavity photon dispersion band and the resonance energy of the optical transition in the intracavity media, which, in our case, is the absorption of P580 dye in liquid crystal solution Mowatt et al. [2010]. It can be seen in Fig.1(c-f) that the blueshift is present when the vertically polarized cavity mode energy at $k_x = 0$ *is around 2.16 eV or higher. Such behaviour is expected assuming the proposed above blueshift mechanism associated with the saturation of the optical transitions, as the P580 dye absorption flattens around 0 below 2.15 eV, while experiencing a strong increase above 2.16 eV Mowatt et al. [2010] (see table in the inset Fig.1(i)), which, upon the saturation of the optical transition, leads to the corresponding decrease of the refractive index and cavity mode renormalization under the optical pumping area.*

Comment #5 (R3C5): *5. The authors state that Frenkel excitons do not support repulsive interactions. In that case, what is the physical origin of the localized potential induced by the tightly focused pump? what is the "potential" in the weak-coupling regime? Could the authors elaborate on how such a potential is defined in a purely photonic lasing system without exciton–exciton interaction? How does this description account for the observed photon outflow from the pumped spot?*

Authors' reply: We thank the referee for the comment. The photonic system studied in this work differs significantly from polariton systems, where the potential is related to the repulsive properties of Wannier-Mott excitons. Although the final interference effect may resemble interactions from polariton systems, the mechanism is not related to polariton-polariton or polariton-exciton interactions. In the case of the system studied, the excitation laser beam saturates the molecular optical transitions as shown by Yagafarov et al. [2020]. This causes a local modification of the refractive index (see also reply to the Comments #2, 3, and 4). In the case of a small exciton spot, a sufficient gradient of the intracavity refractive index is present due to the saturation effect mentioned above, acting as a spatially localized "potential" for the photons propagating away from the excitation spots. This gradient is

sufficient to cause the outflow of photons from the excitation region, thus imparting momentum to the photons in the cavity plane. To quantitatively describe this effect, we employ a set of non-Hermitian 2D Schrödinger equations (Sec. 4.3), representing a paraxial limit of Maxwell-Bloch equations, containing a phenomenologically introduced blueshift parameter (G), which, together with the spatially defined pump profile, represents an analog of potential in a standard Schrödinger equation picture. Such treatment results in a refractive index playing the role of an effective potential for photons, with areas of lower refractive index acting similarly to a repulsive potential and areas of high refractive index acting similarly to a potential well Joannopoulos et al. [2008]. The observed effect of photons gaining in-plane momentum and ring-shaped distribution in Fourier space is described in detail in reply to Comment #2. We also note that the effective treatment of the alternating refractive index as a "potential" for photons is widely adopted in photonic crystal physics Joannopoulos et al. [2008].

To help readers better understand the concept in question, we introduced a series of changes, reflecting our replies to Comments 2-5, in Sec. 2.1 of the main text.

Comment #6 (R3C6): *6. The authors repeatedly mention "room-temperature" and "weak coupling regime," because they employ the concept of polariton studies. However, this seems to dilute rather than strengthen the novelty of the work. Conventional GaAs-based VCSELs already demonstrate high-quality lasing at room temperature. And since author's LCMC system does not involve polariton lasing or cryogenic conditions, it is unclear why these aspects need to be highlighted. Could the authors better justify the necessity of emphasizing room-temperature and weak-coupling operation in this context?*

Authors' reply: We thank the reviewer for allowing us to expand our justification and explain this point. We agree that high-quality room-temperature lasing has indeed been demonstrated in conventional GaAs VCSEL systems. However, in-plane phase-locking between separately pumped arrays of conventional VCSELs remains unattainable, since it requires sophisticated approaches to introduce feedback mechanisms between arrays of lasers and are neither easily tunable nor reconfigurable Xie et al. [2020], Noda et al. [2024]. In-plane phase-locking was, however, achieved in polariton lasing systems based on similar (GaAs) samples, but usually requiring cryogenic temperatures to operate and even more effort to fabricate and remain in the strong coupling regime. It should be specially noted that conventional systems, such as GaAs, require very advanced growth technologies, such as molecular beam epitaxy (MBE) or metalorganic vapour-phase epitaxy (MOVPE), which leads to high costs in obtaining the structure. In this sense, our liquid crystal-organic microcavity technology aims to reduce these systems' manufacturing costs and technological limitations, achieving a highly-tunable and low-cost structure operating at room temperature.
In this paper, we particularly emphasize the importance of the weak coupling regime for two reasons. First reason concerns the novelty of the reported effects and the underly-

ing physics. To avoid confusion for the reader, we want to clearly distinguish our organic LCMC platform from polaritonic systems operating in the strong coupling regime, as some of the effects hereby reported have been observed with polaritonic Bose-Einstein condensates: phase-locking between the condensates was reported previously Ohadi et al. [2016], Tao et al. [2022], as well as next-nearest-neighbour regime of coupling between the optical lattice cites Dovzhenko et al. [2023]. In our case, however, the underlying physical mechanisms for the in-plane propagation carrying phase information, the corresponding establishment of mutual coherence, and non-trivial coupling regime are significantly different despite the visual similarities. The second reason, and also a potential technological benefit of the presented system, is that strong coupling conditions are typically more challenging to maintain at high temperatures, which means that efficiently operating devices based on strong coupling that are robust against photobleaching and degradation remain relatively rare due to the need for cooling. Therefore, the easier availability of weak coupling highlighted here enables reductions in both fabrication and operational costs. It's also worth noting that the integration of the dye with the liquid crystals in our microcavity system plays a significant role, as it enables extensive tunability of the phase-locked arrays of emitters via applied electric field (voltage), unattainable with condensates of polaritons. This tunability allows us to demonstrate switchable operation of the phase-locking mechanism and explore, for example, the Rashba–Dresselhaus spin–orbit coupling regime, which is inaccessible in conventional platforms due to the inherent limitations of fully solid-state systems with intrinsic properties.
To underpin the novelty of our study, we summarize the following major aspects reported in our weakly-coupled platform at room temperature: (i)the realization of ballistically coupled and optically reconfigurable arrays of coherent emitters in the weak coupling regime, (ii) unique electrical tunability of in-plane coupling, (iii) directionally dependent spin-resolved coupling between spatially separated lasing states in the Rashba-Dresselhaus spin-orbit coupling regime, and (iv) demonstration of the next-nearest-neighbor coupling at room temperature.

Thus, we introduce a new physical platform and approach in the context of optical lattice physics, which is based on new physical phenomena, offers significant technological benefits over existing solutions, and is capable of entering otherwise unattainable regimes of operation. To highlight these key advantages over existing solutions based on both conventional VCSELs and polaritonic systems, we emphasize the weak coupling regime and room-temperature operation contextualized within our proposal. To address these points, we have added changes to the introduction section of the manuscript.

Comment #7 (R3C7): *7. The definition of the radius k_l is unclear. Please specify its meaning and indicate it in figures.*

Authors' reply: We thank the referee for allowing us to clarify this point.
The term k_l is the averaged absolute value of the in-plane photon outflow wavenumber.

Hence, for the case of angle-independent emission, k_l corresponds to the radius of the ring in Fourier space and characterizes the momentum related to photons propagating in-plane. We added the following definition of k_l to the last paragraph of Sec 2.1, where it first appears:

We define k_l as an averaged absolute value of the in-plane photon outflow wavenumber, which for the angle-independent emission in $k_x - k_y$ plane corresponds to the radius of the ring-shaped emission in Fourier space (Fig.1(c)).

To help readers better understand the role of k_l, we added a guide-to-eye line to the momentum space in Fig.1(c) and the following information to the figure caption: *Grey dashed ring in momentum-space in* **(c)** *with the radius (k_l) corresponds to the average value of the in-plane photon outflow wavenumber.*

Comment #8 (R3C8): *8. In Fig. 1(c–e), the dashed lines make the PL features difficult to see; adjusting their color or thickness would improve the data visibility.*

Authors' reply: We agree with the referee that the dashed lines make the PL features difficult to see. To improve data visibility, we decreased the thickness of the dashed lines in the corresponding figure.

Comment #9 (R3C9): *9. The sentence "It has been shown [48, 49] that, in organic systems, the blueshift primarily arises from the saturation of molecular transitions and the associated refractive index change and hence, is present in both weak and strong light-matter coupling regimes and, importantly, is wavelength dependent, see Fig 1(c,d,e)." is difficult to follow due to its length and multiple clauses.*

Authors' reply: We thank the reviewer for carefully reading our manuscript and suggesting this correction, which will improve the clarity of our work.

The sentence quoted by the reviewer will read as follows in the new version of the manuscript:

It has been shown [48, 49] that, in organic systems, the blueshift primarily arises from the saturation of molecular transitions and the associated refractive index change. Hence, it is present in both weak and strong light-matter coupling regimes and, importantly, is wavelength-dependent, see Fig.1(c-f).

Comment #10 (R3C10): *10. It is unclear whether the term "supermode" is newly introduced in this work or adopted from existing literature. If it is a new concept, please provide a clear definition. Otherwise, a proper reference should be cited when the term first appears.*

Authors' reply: We thank the referee for this suggestion. We define the lasing supermode as a spatially extended coherent lasing state consisting of two (dyad supermode), three (triad supermode), or more phase-locked spatially separated and individually pumped lasing spots, which are coupled via in-plane coherent photon exchange. This is the adaptation of the more common definition of a supermode as an optical state or collective mode formed as a result of optical coupling between the fields of individual coherently emitting elements of an array.

In the original manuscript, we explain our understanding of the "supermode" term within the following sentence:

An organic liquid crystal-filled microcavity is introduced as a new material platform where a spatially extended coherent lasing state, or "supermode", appears due to the blueshift-induced near-field transverse coupling between distinct spatially pumped states.

Following the reviewer's suggestion, we decided to alter the text by adding a more explicit definition and references [Paoli et al. [1984], Mehuys et al. [1988], Ginis et al. [2023]]. The corresponding fragment in the new version of the introduction reads:

The term "supermode" [Paoli et al. [1984], Mehuys et al. [1988], Ginis et al. [2023]] is used here to refer to the spatially extended coherent lasing state consisting of two or more phase-locked spatially separated and individually pumped lasing spots, which are coupled via in-plane coherent photon exchange.

Comment #11 (R3C11): *Figure 3 appears somewhat disconnected from the main flow of the manuscript. It might improve readability if this figure is moved after Figure 6 or placed in the Supplementary Information. (This is just a suggestion.)*

Authors' reply: We appreciate the reviewer's thoughtful comment, and we understand that the data in Figure 3 goes beyond the one-dimensional configuration of excited spots presented in the rest of the manuscript. However, we believe that it supports one of the key results of this work, which is the full optical reconfigurability of extended lasing states in a 2D plane of the cavity and, therefore, is an integral part of our story. With this figure, we prove the ability to arbitrarily locate the pumping spots and reconfigure the supermode's interference pattern using a spatial light modulator. This feature directly extends the possibilities of the one-dimensional dyad configuration presented in Figure 2 to a two-dimensional lattice of coupled lasing states. We show Figure 3 particularly in Sec. 2.2 to demonstrate the isotropy of the system in the 2D plane in the regime of zero external voltage, where in-plane momentum is angle-independent, and the coupling is possible in any direction. Meanwhile, up to this point of the manuscript, we focused only on optical modulation using a spatial light modulator to alter the spatial position of the lasing spots, while other parameters, such as the voltage applied to the sample or the laser polarization, remained fixed. The following

sections of the manuscript concern the electrical control of the coupling and coupling control using the polarization of the excitation beam. We believe that moving this figure to the end of the manuscript would disrupt the order of our story, where we present the first possibility of reconfigurability using spatial light modulation (number of spots and distance between spots), then prove the system's tunability via the properties of the sample (electrical control over coupling in a dyad lasing supermode) and the polarization pattern of the exciting beam (unconventional coupling in 1D lasing supermode).

Reviewer #4 (Remarks to the Author):

I co-reviewed this manuscript with one of the reviewers who provided the listed reports. This is part of the Nature Communications initiative to facilitate training in peer review and to provide appropriate recognition for Early Career Researchers who co-review manuscripts.

Authors' reply We thank the reviewer for their input in co-reviewing the manuscript.

References

Renjie Tao, Kai Peng, Louis Haeberlé, Quanwei Li, Dafei Jin, Graham R Fleming, Stéphane Kéna-Cohen, Xiang Zhang, and Wei Bao. Halide perovskites enable polaritonic XY spin Hamiltonian at room temperature. *Nature Materials*, 21(7):761–766, jul 2022. ISSN 1476-1122. doi: 10.1038/s41563-022-01276-4. URL https://www.nature.com/articles/s41563-022-01276-4.

Kai Peng, Wei Li, Natalia G. Berloff, Xiang Zhang, and Wei Bao. Room temperature polaritonic soft-spin XY Hamiltonian in organic–inorganic halide perovskites. *Nanophotonics*, 13(14):2651–2658, jun 2024. ISSN 2192-8614. doi: 10.1515/nanoph-2023-0818. URL https://doi.org/10.1515/nanoph-2023-0818https://www.degruyter.com/document/doi/10.1515/nanoph-2023-0818/html.

S. Alyatkin, H. Sigurdsson, A. Askitopoulos, J. D. Töpfer, and P. G. Lagoudakis. Quantum fluids of light in all-optical scatterer lattices. *Nature Communications*, 12(1):5571, sep 2021. ISSN 2041-1723. doi: 10.1038/s41467-021-25845-4. URL http://dx.doi.org/10.1038/s41467-021-25845-4https://www.nature.com/articles/s41467-021-25845-4.

Vishwa Pal, Simon Mahler, Chene Tradonsky, Asher A. Friesem, and Nir Davidson. Rapid fair sampling of the XY spin Hamiltonian with a laser simulator. *Physical Review Research*, 2(3): 1–8, 2020. ISSN 26431564. doi: 10.1103/PhysRevResearch.2.033008.

Marcin Muszyński, Mateusz Król, Katarzyna Rechcińska, Przemysław Oliwa, Mateusz Kędziora, Karolina Łempicka-Mirek, Rafał Mazur, Przemysław Morawiak, Wiktor Piecek, Przemysław

Kula, Pavlos G. Lagoudakis, Barbara Piętka, and Jacek Szczytko. Realizing Persistent-Spin-Helix Lasing in the Regime of Rashba-Dresselhaus Spin-Orbit Coupling in a Dye-Filled Liquid-Crystal Optical Microcavity. *Physical Review Applied*, 17(1):014041, jan 2022. ISSN 2331-7019. doi: 10.1103/PhysRevApplied.17.014041. URL https://link.aps.org/doi/10.1103/PhysRevApplied.17.014041.

Karolina Łempicka-Mirek, Mateusz Król, Luisa De Marco, Annalisa Coriolano, Laura Polimeno, Ilenia Viola, Mateusz Kędziora, Marcin Muszyński, Przemysław Morawiak, Rafał Mazur, Przemysław Kula, Wiktor Piecek, Piotr Fita, Daniele Sanvitto, Jacek Szczytko, and Barbara Piętka. Electrical polarization switching of perovskite polariton laser. *Nanophotonics*, pages 1–8, nov 2024. ISSN 21928614. doi: 10.1515/nanoph-2023-0829. URL http://arxiv.org/abs/2211.11852http://dx.doi.org/10.1515/nanoph-2023-0829.

Katarzyna Rechcińska, Mateusz Król, Rafał Mazur, Przemysław Morawiak, Rafał Mirek, Karolina Łempicka, Witold Bardyszewski, Michał Matuszewski, Przemysław Kula, Wiktor Piecek, Pavlos G. Lagoudakis, Barbara Piętka, and Jacek Szczytko. Engineering spin-orbit synthetic hamiltonians in liquid-crystal optical cavities. *Science*, 366(6466):727–730, November 2019. ISSN 1095-9203. doi: 10.1126/science.aay4182. URL http://dx.doi.org/10.1126/science.aay4182.

Dmitriy Dovzhenko, Denis Aristov, Lucinda Pickup, Helgi Sigurðsson, and Pavlos Lagoudakis. Next-nearest-neighbor coupling with spinor polariton condensates. *Physical Review B*, 108 (16):L161301, oct 2023. ISSN 2469-9950. doi: 10.1103/PhysRevB.108.L161301. URL https://link.aps.org/doi/10.1103/PhysRevB.108.L161301.

S. Alyatkin, J. D. Töpfer, A. Askitopoulos, H. Sigurdsson, and P. G. Lagoudakis. Optical Control of Couplings in Polariton Condensate Lattices. *Physical Review Letters*, 124(20):207402, may 2020. ISSN 0031-9007. doi: 10.1103/PhysRevLett.124.207402. URL https://doi.org/10.1103/PhysRevLett.124.207402https://link.aps.org/doi/10.1103/PhysRevLett.124.207402.

S. Tsesses, E. Ostrovsky, K. Cohen, B. Gjonaj, N. H. Lindner, and G. Bartal. Optical skyrmion lattice in evanescent electromagnetic fields. *Science*, 361(6406):993–996, sep 2018. ISSN 0036-8075. doi: 10.1126/science.aau0227. URL https://www.science.org/doi/10.1126/science.aau0227.

Lechen Yang, Guangrui Li, Xiaomei Gao, and Ling Lu. Topological-cavity surface-emitting laser. *Nature Photonics*, 16(4):279–283, apr 2022. ISSN 1749-4885. doi: 10.1038/s41566-022-00972-6. URL https://www.nature.com/articles/s41566-022-00972-6.

Simon Mahler, Eran Bernstein, Sagie Gadasi, Geva Arwas, Asher A. Friesem, and Nir Davidson. Programmable all optical spin simulator with artificial gauge fields. pages 1–6, aug 2024. URL http://arxiv.org/abs/2408.13865.

Midya Parto, William Hayenga, Alireza Marandi, Demetrios N. Christodoulides, and Mercedeh Khajavikhan. Realizing spin Hamiltonians in nanoscale active photonic lattices. *Nature Materials*, 19(7):725–731, jul 2020. ISSN 1476-1122. doi: 10.1038/

s41563-020-0635-6. URL http://dx.doi.org/10.1038/s41563-020-0635-6https://www.nature.com/articles/s41563-020-0635-6.

Ying Zuo, Bohan Li, Yujun Zhao, Yue Jiang, You-Chiuan Chen, Peng Chen, Gyu-Boong Jo, Junwei Liu, and Shengwang Du. All-optical neural network with nonlinear activation functions. *Optica*, 6(9):1132, sep 2019. ISSN 2334-2536. doi: 10.1364/OPTICA.6.001132. URL https://opg.optica.org/abstract.cfm?URI=optica-6-9-1132.

Zaijun Chen, Alexander Sludds, Ronald Davis, Ian Christen, Liane Bernstein, Lamia Ateshian, Tobias Heuser, Niels Heermeier, James A. Lott, Stephan Reitzenstein, Ryan Hamerly, and Dirk Englund. Deep learning with coherent VCSEL neural networks. *Nature Photonics*, 17 (8):723–730, aug 2023. ISSN 1749-4885. doi: 10.1038/s41566-023-01233-w. URL https://www.nature.com/articles/s41566-023-01233-w.

M. Matuszewski, A. Prystupiuk, and A. Opala. Role of all-optical neural networks. *Phys. Rev. Appl.*, 21:014028, Jan 2024. doi: 10.1103/PhysRevApplied.21.014028. URL https://link.aps.org/doi/10.1103/PhysRevApplied.21.014028.

Thomas L. Paoli, William Streifer, and Robert D. Burnham. Observation of supermodes in a phase-locked diode laser array. *Applied Physics Letters*, 45(3):217–219, 08 1984. ISSN 0003-6951. doi: 10.1063/1.95188. URL https://doi.org/10.1063/1.95188.

D. Mehuys, K. Mitsunaga, L. Eng, W. K. Marshall, and A. Yariv. Supermode control in diffraction-coupled semiconductor laser arrays. *Applied Physics Letters*, 53(13):1165–1167, 09 1988. ISSN 0003-6951. doi: 10.1063/1.100010. URL https://doi.org/10.1063/1.100010.

Vincent Ginis, Ileana-Cristina Benea-Chelmus, Jinsheng Lu, Marco Piccardo, and Federico Capasso. Resonators with tailored optical path by cascaded-mode conversions. *Nature Communications*, 14(1):495, Jan 2023. ISSN 2041-1723. doi: 10.1038/s41467-023-35956-9. URL https://doi.org/10.1038/s41467-023-35956-9.

M. H. Chang, M. J. Frampton, H. L. Anderson, and L. M. Herz. Intermolecular Interaction Effects on the Ultrafast Depolarization of the Optical Emission from Conjugated Polymers. *Physical Review Letters*, 98(2):027402, jan 2007. ISSN 0031-9007. doi: 10.1103/PhysRevLett.98.027402. URL https://link.aps.org/doi/10.1103/PhysRevLett.98.027402.

A. J. Musser, S. K. Rajendran, K. Georgiou, L. Gai, R. T. Grant, Z. Shen, M. Cavazzini, A. Ruseckas, G. A. Turnbull, I. D. W. Samuel, J. Clark, and D. G. Lidzey. Intermolecular states in organic dye dispersions: excimers vs. aggregates. *Journal of Materials Chemistry C*, 5(33):8380–8389, 2017. ISSN 2050-7526. doi: 10.1039/C7TC02655B. URL https://xlink.rsc.org/?DOI=C7TC02655B.

Timur Yagafarov, Denis Sannikov, Anton Zasedatelev, Kyriacos Georgiou, Anton Baranikov, Oleksandr Kyriienko, Ivan Shelykh, Lizhi Gai, Zhen Shen, David Lidzey, and Pavlos Lagoudakis. Mechanisms of blueshifts in organic polariton condensates. *Communications Physics*, 3(1):18, Jan 2020. ISSN 2399-3650. doi: 10.1038/s42005-019-0278-6. URL https://doi.org/10.1038/s42005-019-0278-6.

Johannes D. Plumhof, Thilo Stöferle, Lijian Mai, Ullrich Scherf, and Rainer F. Mahrt. Room-temperature Bose–Einstein condensation of cavity exciton–polaritons in a polymer. *Nature Materials*, 13(3):247–252, mar 2014. ISSN 1476-1122. doi: 10.1038/nmat3825. URL http://www.ncbi.nlm.nih.gov/pubmed/24651424http://www.nature.com/articles/nmat3925http://www.nature.com/articles/nmat3825.

Tomáš Neuman and Javier Aizpurua. Origin of the asymmetric light emission from molecular exciton–polaritons. *Optica*, 5(10):1247, oct 2018. ISSN 2334-2536. doi: 10.1364/OPTICA.5.001247. URL https://www.osapublishing.org/abstract.cfm?URI=optica-5-10-1247.

Anton D. Putintsev, Kirsty E. McGhee, Denis Sannikov, Anton V. Zasedatelev, Julian D. Töpfer, Till Jessewitsch, Ullrich Scherf, David G. Lidzey, and Pavlos G. Lagoudakis. Controlling the spatial profile and energy landscape of organic polariton condensates in double-dye cavities. *Phys. Rev. Lett.*, 131:186902, Oct 2023. doi: 10.1103/PhysRevLett.131.186902. URL https://link.aps.org/doi/10.1103/PhysRevLett.131.186902.

T. J. Kippenberg, S. M. Spillane, and K. J. Vahala. Modal coupling in traveling-wave resonators. *Opt. Lett.*, 27(19):1669–1671, Oct 2002. doi: 10.1364/OL.27.001669. URL https://opg.optica.org/ol/abstract.cfm?URI=ol-27-19-1669.

J. D. Töpfer, H. Sigurdsson, L. Pickup, and P. G. Lagoudakis. Time-delay polaritonics. *Communications Physics*, 3(1), January 2020. ISSN 2399-3650. doi: 10.1038/s42005-019-0271-0. URL http://dx.doi.org/10.1038/s42005-019-0271-0.

Yao Li, Xuekai Ma, Xiaokun Zhai, Meini Gao, Haitao Dai, Stefan Schumacher, and Tingge Gao. Manipulating polariton condensates by Rashba-Dresselhaus coupling at room temperature. *Nature Communications*, 13(1):3785, dec 2022. ISSN 2041-1723. doi: 10.1038/s41467-022-31529-4. URL https://www.nature.com/articles/s41467-022-31529-4.

Carrie Mowatt, Stephen M. Morris, Myoung Hoon Song, Timothy D. Wilkinson, Richard H. Friend, and Harry J. Coles. Comparison of the performance of photonic band-edge liquid crystal lasers using different dyes as the gain medium. *Journal of Applied Physics*, 107(4), feb 2010. ISSN 0021-8979. doi: 10.1063/1.3284939. URL https://pubs.aip.org/jap/article/107/4/043101/896976/Comparison-of-the-performance-of-photonic-band.

John D. Joannopoulos, Steven G. Johnson, Joshua N. Winn, and Robert D. Meade. *Photonic Crystals: Molding the Flow of Light (Second Edition)*. Princeton University Press, 2 edition, 2008. ISBN 0691124566. URL http://www.amazon.com/Photonic-Crystals-Molding-Light-Second/dp/0691124566%3FSubscriptionId%3D13CT5CVB80YFWJEPWS02%26tag%3Dws%26linkCode%3Dxm2%26camp%3D2025%26creative%3D165953%26creativeASIN%3D0691124566.

Yi-Yang Xie, Pei-Nan Ni, Qiu-Hua Wang, Qiang Kan, Gauthier Briere, Pei-Pei Chen, Zhuang-Zhuang Zhao, Alexandre Delga, Hao-Ran Ren, Hong-Da Chen, Chen Xu, and Patrice Genevet. Metasurface-integrated vertical cavity surface-emitting lasers for programmable directional lasing emissions. *Nature Nanotechnology*, 15(2):125–130, feb 2020. ISSN 1748-3387. doi:

10.1038/s41565-019-0611-y. URL http://dx.doi.org/10.1038/s41565-019-0611-yhttps://www.nature.com/articles/s41565-019-0611-y.

Susumu Noda, Masahiro Yoshida, Takuya Inoue, Menaka De Zoysa, Kenji Ishizaki, and Ryoichi Sakata. Photonic-crystal surface-emitting lasers. *Nature Reviews Electrical Engineering*, 1(12):802–814, nov 2024. ISSN 2948-1201. doi: 10.1038/s44287-024-00113-x. URL http://dx.doi.org/10.1038/s44287-024-00113-xhttps://www.nature.com/articles/s44287-024-00113-x.

H. Ohadi, R. L. Gregory, T. Freegarde, Y. G. Rubo, A. V. Kavokin, N. G. Berloff, and P. G. Lagoudakis. Nontrivial Phase Coupling in Polariton Multiplets. *Physical Review X*, 6(3):031032, aug 2016. ISSN 2160-3308. doi: 10.1103/PhysRevX.6.031032. URL https://link.aps.org/doi/10.1103/PhysRevX.6.031032.
